# Intraoperative Mass Spectrometry in Oncology: Technologies, Clinical Applications, and Challenges

**DOI:** 10.3390/molecules31081287

**Published:** 2026-04-15

**Authors:** Robert Onulov, Marius Georgescu, Corina Flangea, Adela Chirita-Emandi, Alina-Florina Serb

**Affiliations:** 1 Doctoral School, Victor Babeş University of Medicine and Pharmacy, Eftimie Murgu Sq. No. 2, 300041 Timisoara, Romania; robert.onulov@umft.ro; 2 Physiology Discipline, Functional Sciences Department, Victor Babeş University of Medicine and Pharmacy, Eftimie Murgu Sq. No. 2, 300041 Timisoara, Romania; 3 Center of Immuno-Physiology and Biotechnologies (CIFBIOTEH), Victor Babeş University of Medicine and Pharmacy, Eftimie Murgu Sq. No. 2, 300041 Timisoara, Romania; 4 Pharmacology Discipline, Biochemistry and Pharmacology Department, Victor Babeş University of Medicine and Pharmacy, Eftimie Murgu Sq. No. 2, 300041 Timisoara, Romania; flangea.corina@umft.ro; 5 Toxicology and Molecular Biology Department, “Pius Brinzeu” County Emergency Hospital, Liviu Rebreanu Boulevard 156, 300723 Timisoara, Romania; 6 Genetics Discipline, Department of Microscopic Morphology, Center of Genomic Medicine, Victor Babeş University of Medicine and Pharmacy, 300041 Timisoara, Romania; adela.chirita@umft.ro; 7 Regional Center of Medical Genetics Timiș, Clinical Emergency Hospital for Children “Louis Țurcanu”, Iosif Nemoianu Street N°2, 300011 Timisoara, Romania; 8 Biochemistry Discipline, Biochemistry and Pharmacology Department, Victor Babeş University of Medicine and Pharmacy, Eftimie Murgu Sq. No. 2, 300041 Timisoara, Romania; aserb@umft.ro

**Keywords:** intraoperative mass spectrometry, REIMS/iKnife, DESI-MS, MasSpec Pen, PIRL-MS, SpiderMass, CUSA/SSI-MS, real-time molecular diagnostics

## Abstract

Surgical precision is critical in oncology, where complete tumor resection while preserving healthy tissue directly influences patient outcomes. Traditional intraoperative diagnostic tools, such as frozen-section analysis, are limited by time constraints, tissue sampling, and interpretative variability. Intraoperative mass spectrometry (MS) has recently emerged as a transformative approach, enabling rapid, label-free molecular profiling of surgical specimens in real time. Several technologies—including Rapid Evaporative Ionization Mass Spectrometry (REIMS, “iKnife”), Desorption Electrospray Ionization (DESI-MS), Matrix-Assisted Laser Desorption/Ionization (MALDI-MS) Imaging, Picosecond InfraRed Laser mass spectrometry (PIRL-MS), and novel devices such as the MasSpec Pen—offer unique strategies for intraoperative tumor characterization. Applications have been demonstrated across multiple cancer types, including brain, breast, gastrointestinal, and urogenital malignancies, where MS can improve margin assessment, tumor classification, and surgical guidance. Beyond its clinical promise, intraoperative MS faces technical and translational challenges, including high infrastructure costs, a lack of standardization, and the need for robust multicenter validation. Integration with artificial intelligence, imaging modalities, and multi-omics approaches may further enhance its clinical utility. This review summarizes current technologies, clinical applications, limitations, and future perspectives of intraoperative MS in oncology, highlighting its potential to reshape surgical oncology practice.

## 1. Introduction

Cancer remains a leading cause of morbidity and mortality worldwide, accounting for nearly 10 million deaths annually according to the World Health Organization. Its heterogeneity, both between and within tumor types, presents significant challenges for early diagnosis, precise surgical resection, and effective treatment. Surgical treatment continues to be a cornerstone of cancer management and, in the case of many solid tumors, represents the only viable curative option [1,2]. Numerous clinical studies have demonstrated that tumor margin status is a critical determinant of local recurrence, disease-free survival, and overall survival across a wide range of tumor types [3,4,5]. Nevertheless, accurately distinguishing malignant tissue from normal or reactive tissue during surgery remains a major clinical challenge, as tumor heterogeneity, infiltrative growth patterns, prior treatments, and anatomical complexity frequently limit the surgeon’s ability to achieve optimal resection with conventional techniques alone [6,7]. To address these challenges, a variety of intraoperative diagnostic and guidance methods have been developed and implemented in oncologic surgery to improve tumor localization, margin assessment, and therapeutic precision in real time.

Traditional methods for intraoperative assessment include frozen-section histopathology (the intraoperative gold standard for margin assessment, but limited by sampling error, processing time, tissue distortion, the need for specialized personnel, and impractical for continuous, whole-margin assessment and less reliable in certain tissues [8,9,10]) and intraoperative imaging techniques. Among the latter, ultrasound is widely used for real-time tumor localization in hepatic, breast, and gynecologic oncology, offering portability and immediate feedback but limited molecular specificity [11], while intraoperative magnetic resonance imaging (iMRI) and computed tomography (CT), which are mostly used in neurosurgery and selected abdominal interventions, provide high-resolution anatomical information and can compensate for tissue shift during resection [12,13]. However, these procedures are expensive and resource-intensive and provide limited molecular-level insight into tumor biology [13]. In addition, optical and fluorescence-based techniques represent another important class of intraoperative tools. Fluorescence-guided surgery using exogenous agents such as 5-aminolevulinic acid (5-ALA) or indocyanine green (ICG) has demonstrated clinical utility in glioma surgery, hepatobiliary oncology, and sentinel lymph node mapping [14,15,16]. More recently, tumor-targeted fluorescent probes and antibody–dye conjugates have been developed to enhance cancer specificity [17]. While these approaches enable real-time visualization of tumor tissue, their effectiveness is influenced by probe pharmacokinetics, tumor uptake, and signal-to-noise ratios, and they are typically limited to predefined molecular targets. Autofluorescence, Raman spectroscopy, and diffuse reflectance spectroscopy have also been explored for intraoperative tissue characterization, offering label-free analysis but often requiring complex instrumentation and interpretation [18,19,20].

In parallel with diagnostic advances, various intraoperative therapeutic technologies have been introduced to complement or enhance surgical resection [21,22,23]. Nevertheless, their effectiveness relies heavily on accurate intraoperative identification of tumor borders and residual malignant tissue [24].

Despite the diversity of available intraoperative techniques, a common limitation across most methods is the lack of direct, real-time molecular information. Since cancer fundamentally involves altered molecular pathways, metabolism, and cellular signaling, there is increasing interest in technologies that can rapidly characterize the biochemical and molecular properties of tissues during surgery [25,26].

In this context, among analytical platforms, mass spectrometry (MS) has been increasingly recognized as a powerful tool for cancer diagnostics and is increasingly incorporated into both research and clinical workflows [27,28,29]. Moreover, intraoperative MS has emerged as a powerful and versatile approach for real-time tissue analysis in oncology, guiding tissue resection [30]. MS enables the detection of a broad range of biomolecules, including lipids, metabolites, peptides, and small proteins, directly from biological tissues with high sensitivity and specificity [31,32]. Cancer-associated alterations in lipid metabolism, energy pathways, and cellular composition generate particular molecular fingerprints that can be exploited for tumor tissue classification and margin assessment [33,34,35].

Traditional analysis of complex biological samples, however, often requires extensive sample preparation and separation methods, particularly chromatography, coupled with MS, which enhances analytical specificity, but significantly prolongs analysis times, rendering it impractical for intraoperative use. The development of ambient ionization mass spectrometry (AIMS) has dramatically accelerated sample analysis, facilitating the translation of MS into the surgical setting [36]. This progress is primarily due to direct tissue analysis, with minimal or no sample preparation required, enabling rapid molecular characterization during procedures.

MS-based intraoperative technologies exploit the distinct molecular composition of cancerous and normal tissues to assist surgeons in guiding tissue resection to confirm disease diagnosis, assist in the removal of cancer tissue, identify regions of normal tissues, and find sites of cancer metastasis, thereby addressing many challenges in tissue identification that frequently occur during surgical procedures [37]. These methods can be grouped into three broad categories according to their workflow and timing relative to tissue sampling: online (real-time), hybrid (near-real-time), and offline (delayed). While each approach has its own advantages and limitations in terms of speed, spatial resolution, molecular coverage, and workflow compatibility, selecting the appropriate method depends on the clinical setup, anatomical site, and specific information required.

Clinical investigations across multiple oncologic specialties—including neurosurgery, breast, colorectal, gynecologic, and head and neck oncology—have demonstrated that intraoperative MS can distinguish malignant from normal tissue with high accuracy and may outperform conventional methods in certain contexts, highlighting their potential to improve intraoperative decision-making and patient outcomes [30,38,39,40,41,42,43]. Additionally, advances in MS technologies toward integrated platforms via user-friendly handheld devices enable faster, more robust MS analyses in clinical settings, with the potential to accelerate workflows and reduce the cost of clinical procedures [44,45]. MS-based approaches can be combined with machine learning algorithms to enable automated tissue classification, decision support, and pattern recognition, further enhancing their clinical potential [46,47].

Despite significant progress, translating intraoperative MS into routine clinical practice remains challenging due to technical, clinical, and regulatory hurdles. Standardization of sampling protocols, robust statistical models for tissue classification, development of validated reference databases, management of inter-patient and intra-tumoral variability, and integration with existing surgical tools are critical for broader clinical adoption [48,49].

**Figure 1:**
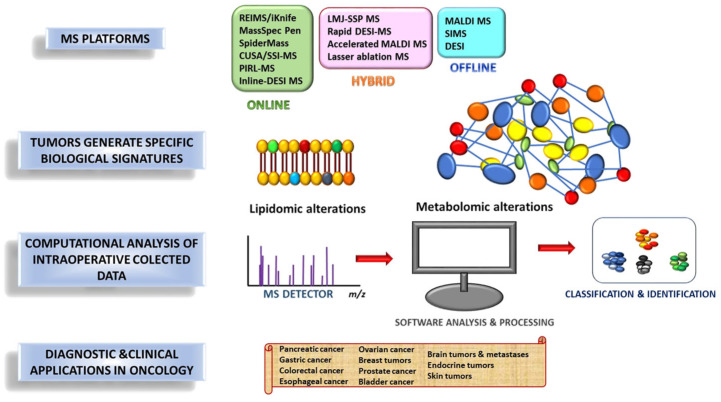
Overview of intraoperative MS methodology in oncologic surgery.

This review provides a comprehensive overview of the developments and applications of intraoperative MS-based methodologies in oncology, within the broader context of intraoperative diagnostic and therapeutic modalities. The main focus is on online approaches, which provide real-time information to support surgical decisions, address the limitations of frozen-section histopathology, and enable continuous assessment of tumor margins while capturing tumor biology beyond morphology alone. The manuscript is organized first to present the main online intraoperative MS technologies, their advantages and limitations, followed by a discussion of the biological signatures detectable in tumors. It then reviews current clinical applications in oncology, across different tumor types, and highlights recent advances in computational approaches for intraoperative MS (Figure 1). Finally, the main barriers to clinical implementation are addressed, together with future perspectives for integrating these technologies into routine practice and advancing precision surgery/medicine.

## 2. Online Intraoperative Mass Spectrometry Technologies in Oncologic Surgery

**Figure 2:**
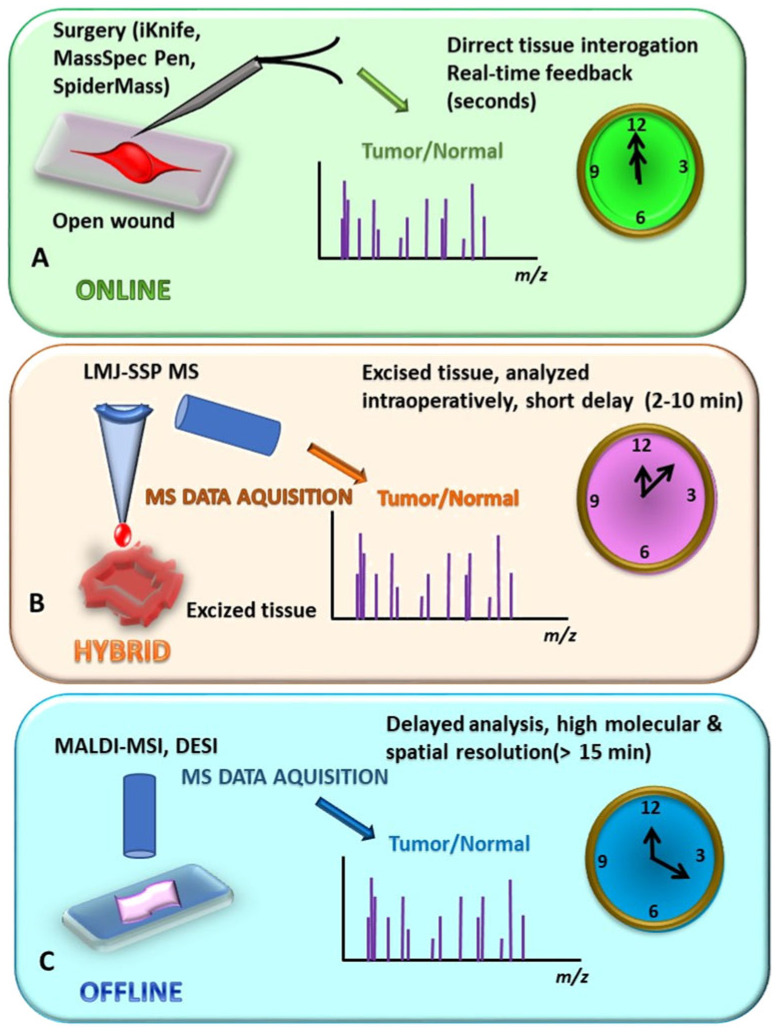
Intraoperative MS-based methodologies. Main characteristics of (**A**) online; (**B**) hybrid, and (**C**) offline approaches.

Intraoperative MS provides rapid molecular analysis of tissues in real-time/near-real-time, offering surgeons chemical information in addition to morphological evaluation. Several technologies have been adapted for clinical use and are grouped into online, offline, or hybrid MS-based methodologies (Figure 2, Table 1). Each technique offers distinct advantages in terms of speed, sensitivity, and ease of integration in the surgical workflow (Table 2). The quantitative values reported in Table 2 (e.g., time to result and spatial resolution) are indicative ranges derived from representative studies in the literature. These parameters depend strongly on the acquisition mode (e.g., point analysis versus imaging), instrument configuration, and clinical workflow implementation. Where variability across studies was significant, qualitative descriptors were preferred over fixed numerical values.

The main obstacle to integrating either MS-based technique into operative practice is the need to work in real time and obtain data as quickly as possible.

Online MS approaches, such as rapid evaporative ionization MS (REIMS, e.g., iKnife), SpiderMass, MassSpecPen, Picosecond Infrared Laser Mass Spectrometry (PIRL-MS), and Cavitron Ultrasonic Surgical Aspirator/Sonic Spray Ionization MS (CUSA/SSI-MS), allow for direct examination of tissues during surgery without the need for tissue excision [38,39,40,41,50,51], managing to mitigate the time constraints seen in earlier MS-based methods. These techniques typically generate a signal within seconds, providing immediate feedback to the surgeon on tissue identity, margin status, or tumor subtype, enabling their application in the clinical workflow (Table 1 and Table 2). Yet, current limitations include the depth of tissue penetration and the extent of tissue damage. Commonly reported issues are restricted penetration depth, preferential effects on certain tissue types, and limited spatial resolution.

Offline MS methods, including Desorption Electrospray Ionization Mass Spectrometry (DESI-MS) imaging and Matrix-Assisted Laser Desorption/Ionization Mass Spectrometry (MALDI-MS) imaging, involve analysis of excised tissue sections with higher spatial and molecular resolution but longer processing times (complex methods of preparation, ranging from tens of minutes to over 30 min) [30,52,53]. These methods enable detailed molecular mapping of tumor margins, subtype identification, and correlation with histopathology, but their delayed feedback limits their immediate utility in guiding real-time surgical decisions (Table 1 and Table 2).

Hybrid MS variants combine features of online and offline analysis. Techniques such as liquid microjunction surface sampling probe MS (LMJ-SSP-MS), rapid DESI-MS, and accelerated MALDI-MS analyze small excised tissue samples within minutes (typically 2–10 min), providing near-real-time information [42,54,55]. These methods offer higher spatial resolution and molecular fidelity compared with fully online approaches, making them suitable for rapid intraoperative margin assessment while maintaining reasonable workflow integration. However, limitations in tissue preparation, workflow coordination, and still longer times until results can be delivered have not yet been fully overcome (Table 1 and Table 2).

Online intraoperative MS methodologies are defined by their ability to deliver immediate or near-immediate molecular information during surgical procedures, without the need for tissue removal, transport, or prolonged preparation. In these approaches, sampling is either continuous or performed directly at the surgical site, and molecular data are generated fast enough to influence surgical decisions in real time. Most online systems rely on ambient ionization and are tightly integrated with surgical instruments or probes.

**Table 1:** Intraoperative MS technologies in oncologic surgery.

Type	MS Technique	Sampling Strategy	Ionization Mode	Primary Molecular Information	Clinical Applications and Maturity *	Ref.
**Online (Real-Time)**	REIMS (iKnife)	Surgical aerosol generated during electrocautery	Thermal desorption with MS detection	Lipids, metabolites	Real-time intraoperative tissue classification and margin guidance in brain, breast, colorectal, gynecologic, and head and neck cancer’ Advanced translational to early clinical implementation	[38,40,56,57,58,59,60,61]
	MassSpec Pen	Gentle liquid microjunction extraction from tissue surface	Solvent-assisted ambient ionization	Lipids, metabolites	In situ tissue intraoperative diagnosis and tumor margin assessment; intraoperative decision support Advanced translational/early clinical stage	[50,62,63,64,65,66]
	Spider Mass	Remote laser ablation plume aspirated via tubing	Infrared laser desorption + MS	Lipids, metabolites	Real-time tissue intraoperative identification, tumor detection and minimally invasive margin assessment Early translational	[40,67,68,69,70,71]
	CUSA/SSI-MS	Surgical aspirate generated by ultrasonic tissue fragmentation	Secondary electrospray ionization of aerosol	Lipids, metabolites	Intraoperative brain tumor characterization during ultrasonic aspiration; exploratory tumor identification Experimental/research	[41,72]
	Inline DESI-MS **	Direct in situ tissue analysis adjacent to the surgical field	Ambient electrospray ionization	Lipids, metabolites	Direct in situ tissue intraoperative interrogation and margin assessment Early translational	[73,74,75,76]
	Laser-based MS (e.g., PIRL-MS)	Laser ablation plume	Soft laser desorption	Metabolites, lipids	Rapid tissue classification; Experimental intraoperative tissue analysis Research/experimental stage	[77,78,79]
**Offline (Intraoperative)**	DESI-MS **	Excised fresh or frozen tissue sections	Ambient electrospray ionization	Lipids, metabolites	Margin assessment; tumor subtype identification; correlation with histopathology Early translational stage	[53,80,81]
	MALDI-MS	Tissue sections with matrix application	Laser desorption/ionization	Peptides, proteins, lipids	Molecular tumor profiling; intraoperative diagnostic support Experimental/research stage	[30,52,53]
	SIMS	Fixed or frozen tissue	Ion beam sputtering	Elements, lipids	High-resolution tissue analysis; mechanistic tumor studies. Experimental/research (no routine clinical implementation)	[82]
**Hybrid/Near-Real-Time**	Rapid DESI-MS **	Fresh tissue samples analyzed immediately after excision (without full histological preparation)	Ambient electrospray ionization	Lipids, metabolites	Rapid margin evaluation; surgical decision support Experimental/research stage	[55]
	Accelerated MALDI-MS	Rapid matrix-assisted tissue analysis	Laser desorption/ionization	Proteins, lipids	Rapid tissue classification; intraoperative pathology support Experimental—frozen brain specimen samples and BC patients, along with mouse tissue samples. Experimental/research stage	[54]
	Laser ablation MS-LAESI MS (hybrid)	Targeted tissue ablation	Laser desorption	Metabolites, lipids	Experimental tumor identification; potential margin assessment Experimental/research	[83]
	LMJ-SSP-MS	Localized liquid microjunction extraction from excised tissue	Liquid microjunction surface sampling probe	Lipids, metabolites, small molecules	Rapid molecular margin analysis; pathology correlation Experimental/research stage	[42]

* Clinical maturity categories (experimental/research, early translational, advanced translational/early clinical, clinical validation, and clinical implementation) are based on representative literature and reflect general stages of development rather than formal/strict regulatory classifications. Factors such as study design (e.g., first-in-human feasibility, prospective intraoperative studies), cohort size (including multicenter validation), and degree of clinical integration were used for clinical maturity categories. These levels were defined as follows: Experimental/research: preclinical (in vitro, ex vivo, or animal) and feasibility studies; Early translational: first-in-human studies or limited intraoperative feasibility; Advanced translational/early clinical: prospective intraoperative studies, typically single-center or limited cohorts; Clinical validation: larger prospective cohorts and/or multicenter validation studies; Clinical implementation: regulatory-cleared devices and/or routine clinical use. Clinical applications are presented in concise form; detailed examples of study design and clinical validation for each technology are provided in the main text; detailed performance characteristics are provided in Table 2 and Supplementary Table S1. ** DESI-MS is presented in multiple configurations depending on the sampling strategy: conventional DESI-MS is applied to excised tissue sections (offline), rapid DESI-MS to freshly collected tissue samples with minimal delay (near-real-time), and inline DESI-MS to direct tissue analysis in proximity to the surgical field (online).

**Table 2:** Advantages, limitations, and typical performance characteristics of intraoperative MS technologies in oncologic surgery.

Type	MS Technique	Advantages	Limitations	Representative Performance (Sensitivity/Specificity) ***	Ref.
**Online (Real-Time)**	REIMS (iKnife)	Seamless surgical integration; no workflow interruption; immediate feedback Time to result *—seconds (typically 1–5 s per real-time intraoperative analysis during electrosurgical sampling, depending on system configuration and data processing) Continuous, real-time feedback No tissue removal required	Limited spatial resolution; thermal degradation; dependent on electrosurgery Low spatial resolution	Sensitivity ~74–99%; specificity ~90–100%	[38,40,56,57,58,59,60,61]
	MassSpec Pen	Non-destructive; hand-held; compatible with open and minimally invasive surgery No tissue removal required Time to result *—seconds (typically ~3–10 s per measurement, including extraction, MS analysis, and data processing) Near-real-time feedback	Point-by-point sampling; limited depth Moderate spatial resolution	Sensitivity ~95–97%; specificity ~90–96%	[50,62,63,64,65,66]
	Spider Mass	Remote, non-contact sampling; minimal thermal damage; compatible with open and endoscopic surgery	Limited penetration depth; system complexity, Moderate spatial resolution	Limited sensitivity/specificity data (Accuracy ~72–83%)	[40,67,68,69,70,71]
	CUSA/SSI-MS	Integrates with neurosurgical standard tools; continuous sampling Near-real-time operative feedback No tissue removal required Time to result *—seconds (continuous analysis of surgical aspirate)	Tissue bias toward softer tissue; limited spatial resolution	Not consistently reported	[41,72,73]
	Inline DESI-MS	Label-free; minimal sample preparation Real-time operative feedback Time until result *—seconds (depending on acquisition time per spot) No tissue removal required	Sensitivity to surface conditions; positioning constraints Moderate spatial resolution	Sensitivity ~89–93%; specificity ~83–100%	[74,75,76,77]
	Laser-based MS (e.g., PIRL-MS)	Minimal thermal damage; high molecular fidelity Near-real-time intraoperative feedback Time until result *—seconds (per laser sampling event) No tissue removal required	Limited availability; complex instrumentation Moderate spatial resolution	Sensitivity ~92–96%; specificity ~96–99% (selected studies)	[78,79,80]
**Offline (Intraoperative)**	DESI-MS	High molecular specificity; spatial mapping; minimal preparation Spatial resolution ** typically ~200 µm in clinical DESI-MSI imaging workflows, depending on acquisition settings (e.g., pixel size and scan parameters) Time until result *—minutes	Not continuous; requires tissue excision Delayed intraoperative feedback	Sensitivity ~88–97%; specificity ~83–100%	[53,81,82]
	MALDI-MS	Excellent spatial resolution; broad molecular coverage Spatial resolution ** ≈ 10–50 µm (MALDI imaging, matrix-assisted tissue sections)	Sample preparation time; workflow complexity Tissue removal required, delayed intraoperative feedback, Time lag (15–45 min) until results	High accuracy reported; sensitivity/specificity not consistently available	[30,52,53]
	SIMS	Subcellular resolution—ultra-high spatial resolution (submicron spatial resolution—SIMS imaging)	Destructive; limited biomolecular range, tissue removal required, time lag until result (tens of minutes) Delayed intraoperative feedback	Not consistently reported	[83]
**Hybrid/Near-Real-Time**	Rapid DESI-MS	Balance of speed and molecular detail, High spatial resolution, near-real-time intraoperative feedback, time until result *—2–5 min (rapid DESI analysis of excised tissue)	Requires workflow coordination Tissue removal required	Not consistently reported (limited standardized metrics)	[55]
	Accelerated MALDI-MS	Very High spatial resolution with reduced delay, time until result *: 15 min (including rapid matrix-assisted analysis workflow)	Still slower than online MS, tissue removal is required	Not consistently reported	[54]
	Laser ablation MS (hybrid)	Flexible integration, near-real-time intraoperative feedback, time until result *—seconds to minutes (depending on acquisition strategy)	Limited clinical validation, Moderate spatial resolution, variable tissue removal required	Not consistently reported	[84]
	LMJ-SSP-MS	High molecular fidelity; spatially resolved sampling, high spatial resolution, near-real-time intraoperative feedback, time until result *: 2–10 min (depending on sampling strategy and number of analyzed points)	Requires tissue excision; probe stabilization	Not consistently reported	[42]

* Reported time-to-result values represent typical ranges from the cited literature rather than best-case performance and may vary depending on acquisition mode, data processing, and clinical workflow implementation. ** Spatial resolution values (applicable to imaging-based techniques such as DESI-MSI and MALDI-MSI) refer to imaging mode rather than single-spot or bulk sampling and depend on pixel size, scan parameters, matrix preparation, and instrument configuration (including analyzer type). For DESI-MSI, cited clinical studies commonly use ~200 µm imaging resolution, whereas MALDI-MSI can reach finer spatial resolution under optimized imaging conditions. *** Representative performance values are derived from reported ranges in the literature and are intended to provide an overview of typical diagnostic performance. Detailed study-level metrics are provided in Supplementary Table S1.

### 2.1. Rapid Evaporative Ionization Mass Spectrometry (REIMS)

REIMS represents an AIMS technique and the most mature and clinically advanced online MS technology [38,56]. REIMS exploits the aerosol (“surgical smoke”) generated during electrosurgical cutting/coagulation of tissue. In this approach, an adapted electrosurgical instrument (monopolar or bipolar diathermy) serves as both the surgical cutting tool and the ion source. The development of the intelligent surgical knife (iKnife) [38] enabled online REIMS setup, allowing for operation in monopolar or bipolar electrosurgical modes, and was also modified to enable REIMS sampling and analysis.

**Figure 3:**
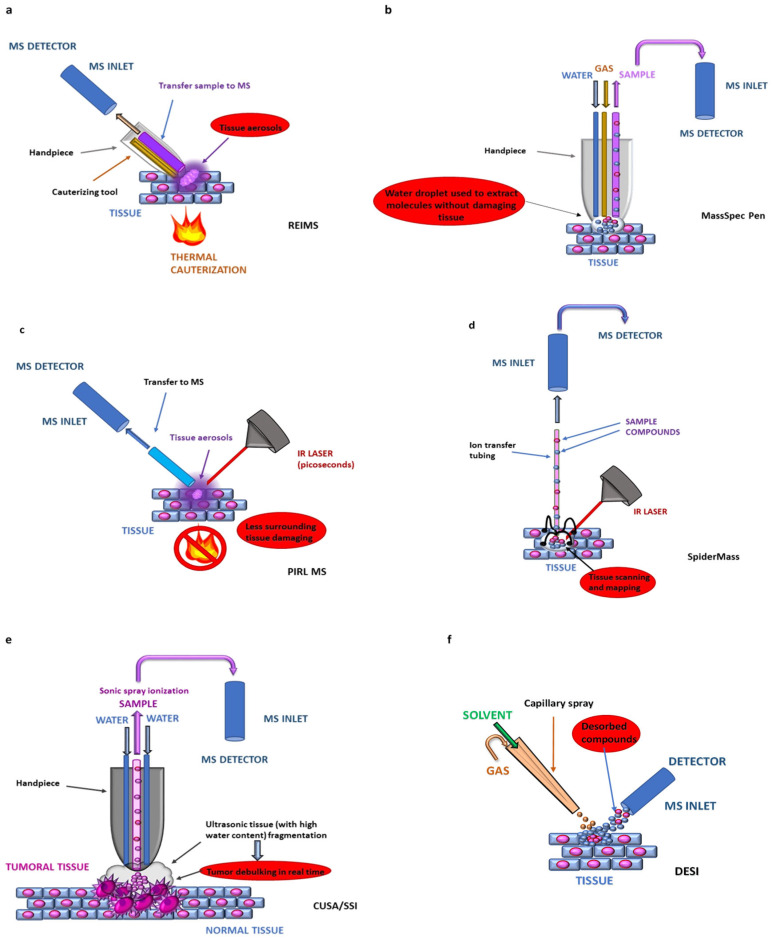
Online intraoperative MS-based methodologies. (**a**) REIMS; (**b**) MassSpecPen; (**c**) PIRL MS; (**d**) SpiderMass; (**e**) CUSA/SSI; (**f**) DESI.

During tissue cutting or coagulation, electrosurgical energy (continuous/pulsed radiofrequency) is applied to tissue. Heat causes intracellular water to rapidly vaporize and cells to burst, releasing molecular constituents (usually lipids and various metabolites) into the aerosol, which is aspirated directly into a mass spectrometer, typically via a transfer line (an atmospheric pressure interface—API), without the need for sample preparation or chromatographic separation (Figure 3a).

The molecular profiles obtained (mass spectra acquired mostly in the *m*/*z* range ~600–1000) are dominated by glycerophospholipids (GPLs), predominantly phosphatidylethanolamines (PEs), phosphatidylcholines (PCs), phosphatidic acids (PAs) and fatty acids (FAs), and other membrane-derived species, which reflect tumor-specific metabolic remodeling and provide a robust discrimination between tumor and normal tissues. Computational algorithms (e.g., Principal component analysis (PCA), linear discriminant analysis (LDA), machine learning algorithms) compare the acquired spectrum against pre-built spectral databases to classify tissue as tumor or normal, and even distinguish histological subtypes and prognostic features in some tumors [38].

The clinical implementation of REIMS using the iKnife has demonstrated continuous, real-time tissue classification with feedback within seconds across multiple cancer types, including breast, colorectal, brain, lung, and gynecological malignancies [56,57,58] (Table S1). Extensive validation studies have shown high concordance between REIMS-based classification and histopathological diagnosis, and strong reproducibility across centers [56], supporting its role in assisting surgical procedures through molecular profiling rather than as a replacement for pathology. In studies on tumor classification, sensitivity and specificity have often exceeded 90% in controlled settings, although values vary by tumor type and clinical state [38,56,57].

As ion generation occurs during tissue cutting and spectral acquisition is continuous, REIMS represents a fully online intraoperative MS approach. The most frequently used intraoperative MS in clinical studies, offering demonstrated high diagnostic accuracy in applications such as breast, colorectal, skin, gynecological, and even brain oncological surgery [38,57,58,59,60,61], as a powerful tool for margin assessment and tissue identification (Table S1).

### 2.2. The MasSpec Pen

MassSpecPen is an MS-based cancer detection and diagnosis system that can be used for both in vivo and ex vivo tissue analysis, which was first reported by Livia S. Eberlin and colleagues in 2017 [50]. It is a solvent-based ambient ionization technique that allows for the analysis of samples distant from the mass spectrometer in a geometry-independent manner, using a handheld device to direct the analysis [50]. In the operating room, the MasSpec Pen device is sterilized and handled by the surgical team like any other handheld instrument. The tip of the “pen” is placed in contact with the surface of the sample. The operator initiates the sampling procedure by pressing an integrated foot pedal, which further activates the delivery of a small aliquot of the solvent (e.g., sterile water ~20 µL) via a polymer tube to the tip of the “pen” in contact with the sample. This results in the extraction of the analytes from the sample into the solvent droplet via a solid–liquid extraction mechanism (≈3 s), and the droplet is then aspirated into a high-resolution mass spectrometer (usually an Orbitrap or similar platform, which is positioned outside the sterile field). Then, the analytes within the solvent droplet are desolvated and ionized via an inlet ionization mechanism, producing diagnostic mass spectra typically within 10–20 s (Figure 3b). Thus, the composition of the extracted molecules allows molecular profiling in seconds without the destructive effects associated with conventional tissue resection tools or extensive preprocessing, which can then be used to predict if the analyzed sample contains malignant cells using machine learning classifiers (such as Lasso regression) and statistical models [62] (Table 2).

The classification of the MasSpec Pen has been debated because it does not provide continuous ion generation during cutting and data streaming. However, when assessed by functional criteria rather than firm temporal continuity, the MasSpec Pen can be reliably included in the online category.

The MasSpec Pen device was developed to assist in the complete excision of cancer during surgery by identifying positive surgical margins during solid tumor resection, providing rapid, nondestructive molecular analysis of tissue surfaces. It was initially used to analyze 253 human tissue biopsies, including normal and cancerous breast, lung, ovary, and thyroid tissues [50]. The mass spectra obtained for each tissue type contained characteristic lipids, metabolites, and some proteins, and the collected data were then used to develop statistical classification models that could accurately discriminate between the normal and cancer samples of each tissue type, with reported accuracies exceeding 94% across tumor types (breast, lung, ovary). Statistical models also allowed differentiation of normal thyroid tissue from papillary thyroid carcinoma with 97.8% accuracy and from follicular thyroid adenoma with 94.7% accuracy. The study also demonstrated the MasSpec Pen’s ability to detect malignant tissue within heterogeneous samples containing intermixed normal and cancerous regions from an ovarian cancer (OC) sample, highlighting its sensitivity to spatial tumor heterogeneity (Table S1).

The performance of the MasSpec Pen for OC diagnosis was further assessed in a 2019 report [63]. The authors analyzed 160 human deidentified ovarian frozen tissue samples, including 78 normal ovaries and 82 serous carcinomas, with the MasSpec Pen and developed classification models to discriminate between the normal and cancer samples with 98.3%, 100.0%, and 92.3% overall accuracy on a training, validation, and test set of samples. Moreover, the report showed the ability of the MasSpec Pen system to distinguish OC from fallopian tube and peritoneum tissue (two of the most common sites for OC metastasis), reaching accuracies of 87.9% and 92.6%, respectively.

In a prospective intraoperative clinical study, the MasSpec Pen was used during 25 BC operations, yielding ≈ 95.9% concordance with postoperative histopathological results across 147 intraoperative analyses. This demonstrates the feasibility and high accuracy of real-time tissue diagnosis for surgical margin evaluation [64].

The MasSpec Pen has also been used to detect pancreatic cancer during excision procedures [65], with both in vivo and ex vivo tissue samples used to distinguish healthy pancreas from pancreatic tumor tissue. It was also used to identify tumor margins near adjacent structures of the pancreas, such as the bile duct. The system was used in 18 pancreatic cancer surgeries, and the collected data enabled the detection of cancerous tissue using MasSpec Pen spectral classifiers, achieving overall agreement with pathology of over 91% and sensitivity and specificity exceeding 90%.

In addition, a large translational study involving the use of the MasSpec Pen in ~100 surgeries in various tissue types (thyroid, parathyroid, lymph nodes, breast, pancreas, bile duct) confirmed its safe integration into standard operating room workflows and rapid molecular profiling capabilities without adverse technical impacts [66].

### 2.3. Picosecond Infrared Laser Mass Spectrometry (PIRL-MS)

PIRL-MS is an ambient ionization technique that uses a picosecond-pulsed mid-infrared laser to desorb and vaporize molecules from the tissue sample with minimal thermal injury (Figure 3c). In the PIRL-MS workflow, a handheld device is positioned near or in gentle contact with the tissue to be sampled, and the laser pulse selectively excites vibrational modes in endogenous water within biological tissues, allowing for rapid ablation with ultra-reduced collateral heat transfer compared with nano- or microsecond lasers—“cold ablation”. This minimizes thermal denaturation of surrounding tissue while generating a plume rich in intact biomolecules (e.g., small metabolites, lipids, and fragments of larger species). The molecules are released directly during ablation, and the ejected plume consists mostly of neutral molecules and clusters. Ionization occurs downstream, typically via atmospheric-pressure chemical ionization (APCI) or by interaction with charged species in the inlet or transfer region of the mass spectrometer. The generated aerosol is then transported through a capillary or transfer line to the mass analyzer for spectral detection, typically via an atmospheric pressure inlet, where ion transmission may be further enhanced by optional electrospray-assisted post-ionization.

Because PIRL does not require an external matrix and causes less thermal damage and less disruption than electrosurgical devices, it has the potential to be applied even in surgical domains where minimizing collateral injury is essential (e.g., neurosurgery). Using intratumoral lipid signatures, PIRL-MS has been shown ex vivo to classify medulloblastoma subgroups with ~99% accuracy, demonstrating the feasibility of rapid molecular subgroup characterization within timeframes compatible with intraoperative decision-making [78]. Another study showed that PIRL-MS could distinguish melanoma, squamous cell carcinoma, and normal skin in mouse models by detecting a set of lipid metabolites as diagnostic biomarkers with high sensitivity and specificity, using just 10 s of data collection [79]. In a large retrospective investigation of spinal tumors, PIRL-MS was used to generate lipidomic profiles, identifying major tumor types, including metastatic carcinoma, schwannoma, and meningioma, with >90% sensitivity and specificity [80] (Table S1).

Although PIRL-MS has not yet been widely adopted in human intraoperative practice, the solid preclinical evidence for rapid, minimally destructive sampling positions it as a promising candidate for future real-time surgical management, especially when computational tissue classification models and spatial registration tools are added.

### 2.4. SpiderMass

SpiderMass was invented and developed by researchers at the PRISM Laboratory at Université de Lille, France, primarily the team led by Philippe Saudemont and Isabelle Fournier [67,69]. The SpiderMass system uses a process termed water-assisted laser desorption/ionization (WALDI) to provide minimally invasive, real-time molecular analysis of biological tissue. A fibered laser is tuned to excite endogenous water (~70–80% in biological tissues) molecules, which act as an in situ matrix equivalent to enable desorption and ionization of tissue biomolecules. Laser-induced vaporization generates a plume of intact molecular ions (lipids, metabolites, small peptides) that is continuously aspirated, in real time, through flexible tubing (several meters) directly into a mass spectrometer analyzer, typically with atmospheric-pressure interfaces, enabling true online data acquisition (Figure 3d). Ogrinc et al. [67] described in their paper how to set up the SpiderMass system for in vivo real-time MS of biological tissues, including instrumentation, coupling to mass analyzers, and data processing.

In another study, Ogrinc et al. [68] extend SpiderMass by coupling the probe to a robotic arm for automated in vivo MS imaging. This study demonstrates 3D topography molecular imaging from biological samples using SpiderMass, highlighting potential future intraoperative and surgical guidance applications. Fatou et al. [40] describe the original SpiderMass instrument, designed for in vivo, real-time MS analysis, demonstrating minimal-damage profiling of biological tissues and their differentiated molecular patterns (e.g., distinguishing cell phenotypes). SpiderMass is presented as the first system for real-time in vivo MS under minimally invasive conditions. The time from ion formation to spectral readout is essentially instantaneous (milliseconds to seconds), providing molecular fingerprints that can be directly compared with reference databases for immediate tissue classification.

Preliminary studies on oral squamous cell carcinoma (OSCC) demonstrated that MS spectra obtained using SpiderMass could differentiate tumor, dysplasia, and peritumoral regions based on lipid metabolic profiles, enabling reliable classification using multivariate statistical models [71] (Table S1).

Moreover, a recent study [70] showed that SpiderMass, combined with machine learning, can be used to analyze glioblastoma tissue in real time and generate molecular fingerprints that diagnose glioblastoma with high accuracy (>90%) after cross-validation, and to stratify isocitrate dehydrogenase (IDH)-wild-type glioblastoma patients into molecular subgroups and prognosis categories with a similar level of performance. Consequently, SpiderMass is a well-suited online intraoperative MS technology for precision oncology in central nervous system (CNS) tumors. However, further validation in larger, multicentric cohorts and standardization of data acquisition and analysis workflows remain necessary before widespread clinical implementation.

### 2.5. CUSA/SSI-MS

CUSA is a surgical instrument widely used in oncologic and neurosurgical procedures [72], which employs a high-frequency ultrasonic tip (typically ~36 kHz) to break soft tissues into small fragments while preserving more fibrous structures, such as nerves and blood vessels. Further, the tissue fragments and the irrigation fluid are aspirated through a suction system, generating a liquefied tissue suspension, which is introduced directly into a mass spectrometer via an ambient ionization interface such as Sonic Spray Ionization (SSI), most commonly a Venturi air jet pump, which nebulizes the aspirated fluid into fine droplets suitable for MS ion generation (Venturi easy ambient sonic-spray ionization—V-EASI interface) [41] (Figure 3e). Thus, in SSI, ion production is achieved by pneumatic nebulization and charge separation in the Venturi-driven spray, based on the biochemical composition of the aspirate, yielding both positive- and negative-ion mass spectra directly from the surgical material. The resulting ions are measured by a mass analyzer (often a time-of-flight or quadrupole system) under atmospheric pressure or via a compatible inlet, producing molecular profiles that reflect the composition of the aspirated tissue and providing real-time molecular characterization. The obtained mass spectra are rich in complex tissue-derived species, particularly lipids (e.g., GPLs, FAs) and multiply charged peptides, in both ion modes, and are comparable in biochemical specificity to other ambient ionization techniques (e.g., REIMS). Preliminary multivariate analyses (e.g., PCA and LDA) confirmed that spectra are tissue-specific, allowing distinction between normal and pathological tissue types such as astrocytoma, meningioma, metastatic tumors, and normal brain or liver tissues in ex vivo models [41]. Thus, the method proved its potential to assist in intraoperative tissue identification during tumor resection, with analyses acquired within seconds of ultrasonic aspiration.

The CUSA/SSI-MS system shares conceptual similarities with REIMS. Still, it is based on mechanical or ultrasonic disruption rather than electrosurgical vaporization, a technique compatible with neurosurgical procedures where ultrasonic aspiration is preferred over electrocautery. CUSA/SSI-MS is recognized as part of ambient ionization MS methods with potential intraoperative applications, particularly in CNS tumor diagnostics [73].

However, achieving consistent ionization efficiency and reproducible aerosol composition remains an important challenge. Moreover, while early results are promising, few large-scale, in vivo clinical studies exist, and a larger validation across tumor types and surgical procedures is needed (Table S1).

### 2.6. Inline DESI-MS

Inline-DESI represents a workflow-optimized adaptation of conventional DESI-MS, which was developed in 2004 by Graham Cooks’ group from Purdue University [74] as an offline ambient ionization technique, enabling real-time/near-real-time analysis (typically within seconds to minutes, depending on the scan parameters and the mass analyzer speed) of fresh tissue during surgery while preserving the fundamental DESI mechanism. The technique preserves tissue integrity and does not require matrix application, vacuum conditions, or extensive preparation [75]. Inline DESI-MS should be distinguished from conventional offline DESI-MS, which is typically applied to excised tissue sections, and from rapid DESI-MS approaches, which analyze freshly collected samples with minimal delay outside the surgical field. In this context, inline DESI-MS is classified as an online technique, as the analysis is performed directly at or adjacent to the surgical field, with minimal sample handling and immediate data acquisition within the intraoperative workflow, typically within seconds to a few minutes from tissue contact to obtaining mass spectra, in contrast to offline workflows that require extended preparation times (Table 2).

In the DESI assay, a fast-moving solvent spray of charged microdroplets (<10 μm in diameter) is directed onto the sample surface, extracting the analytes, which are then desorbed as secondary droplets containing analyte ions [75] (Figure 3f). The resulting ions travel through the air into the atmospheric-pressure interface (transfer tubes), which is connected to the mass spectrometer inlet. The adaptation for inline intraoperative use involves positioning the DESI ionization sprayer (mounted or hand-held) near the exposed tissue surface and the mass spectrometer interface adjacent to the surgical field, so that fresh tissue can be analyzed quickly with minimal sample transport and without extensive preparation. The obtained mass spectra reflect the molecular composition (predominantly lipids and small metabolites) of the tissue in quasi-real time.

Although DESI-MS has been widely used as a research method for ex vivo tissue imaging, the approach using inline sampling during resection showed to be suitable for margin assessment in cancers where biochemical signatures differ markedly between tumor and normal tissues (e.g., lipidomic profiles in gliomas versus adjacent gray and white matter), especially when combined with multivariate statistical models that correlate spectral features with pathology.

Thus, Pirro et al. [76] showed that intraoperative DESI-MS enabled assessment of glioma infiltration and tumor cell percentage (TCP) at margins (sensitivity ~93%, specificity ~83%). Intraoperative mobile DESI-MS systems have been developed to determine IDH mutation status, estimate tumor cell infiltration, and classify disease state from tissue biopsies obtained during surgery in glioma [77].

## 3. Biological Signatures in Tumors

Biological signatures are a series of changes in cancerous tissue that result from structural and functional modifications during malignant transformation.

In tumors, biological signatures relevant to intraoperative MS include molecular alterations detectable in fresh tissue, which can support rapid decisions during surgery. In practice, the most clinically useful intraoperative signatures are mainly lipidomic and selected metabolomic classes, while proteomic markers are currently constrained by sample preparation, acquisition time, and platform compatibility.

### 3.1. Proteomic Alterations

Cancerous tissue can be identified and characterized with increasing accuracy by modern MS techniques that are constantly evolving. Generally, tumors are characterized by extensive proteomic remodeling that reflects both genetic alterations and adaptive responses to the tumor microenvironment: changes in protein expression, modification, and turnover that drive malignant behavior.

Proteomic changes are biologically important in cancer and can reflect tumor type, subtype, and signaling state. Protein analysis by MS can be largely divided into bottom-up and top-down approaches [85]. However, the translation of proteomic alterations into intraoperative MS remains more limited than lipidomic or selected metabolomic signatures. In practical terms, protein-level analysis often requires more extensive sample preparation, may involve digestion-based workflows, and is less compatible with the speed and simplicity required to support real-time intraoperative decisions.

For intraoperative applications, proteomic information is therefore currently more relevant in ex vivo or near-real-time approaches, particularly in MALDI-MS imaging of tissue sections, than in ambient platforms such as REIMS or DESI-MS. Peptides, small proteins, and selected post-translationally modified species may support tissue classification in these workflows. Still, robust intraoperative implementation remains constrained by preparation time, ionization efficiency for higher-mass analytes, and workflow integration.

Thus, proteomic signatures are better viewed as complementary molecular information rather than the main molecular basis of current intraoperative MS applications. In contrast, lipid classes and selected small metabolites remain the most practical analyte groups for rapid assessment of margin status, tumor subtype, and selected molecular alterations in fresh tissue.

### 3.2. Lipidomic Alterations

In addition to proteomic markers, there are obvious alterations in the composition of tissue lipids, from simple to complex lipids.

Alterations in **fatty acid (FA)** composition are consistently observed in tumor tissues and reflect metabolic reprogramming and membrane remodeling. In general, tumors exhibit a shift toward increased levels of long- and very-long-chain FAs, along with enhanced de novo lipogenesis and desaturation activity [86,87] (Table 3). For example, colorectal cancer (CRC) has been associated with decreased levels of short-chain FAs and increased levels of long-chain FAs, such as palmitic acid (16:0) and linoleic acid (18:2) [86].

Several tumor types, including ovarian clear cell carcinoma (OCCC) and clear cell renal cell carcinoma (ccRCC), show enrichment in monounsaturated (MUFAs) and polyunsaturated fatty acids (PUFAs), together with increased fatty acid desaturation indices, reflecting elevated stearoyl-CoA desaturase activity and altered membrane fluidity [88,89,90]. These changes are also reflected in phospholipid composition, where altered incorporation of FA chains into phosphatidylcholine (PC) species has been reported [91].

Although some studies have shown circulating FA alterations in serum or plasma [92,93,94], intraoperative MS primarily captures these changes at the tissue level, where FA-derived signals contribute to broader lipid profiles dominated by phospholipids and related species. In MS-based approaches such as DESI-MS, REIMS, and MALDI-MSI, FAs are therefore typically detected as part of complex lipid profiles that enable discrimination between tumor and normal tissue.

**Ceramide** and wider sphingolipid alterations are consistently observed in tumor tissues and reflect changes in apoptosis, membrane organization, and cell signaling. Particular ceramide species, including long- and very-long-chain variants such as C16:0 and C24:1, have been reported to vary across tumor types and may correlate with tumor progression and biological behavior [95,96]. In addition, shifts in the balance between ceramides and sphingosine-1-phosphate (S1P), often favoring S1P, are associated with pro-proliferative signaling and poorer prognosis [97] (Table 3). In the context of intraoperative MS, these ceramide alterations are typically not used as single biomarkers but contribute to broader sphingolipid patterns relevant for tissue classification and tumor margin assessment.

Complex **glycosphingolipids (GSLs),** including gangliosides, are frequently altered in cancer and are often correlated with aggressive or metastatic disease phenotypes. These changes reflect shifts in sphingolipid metabolism that affect apoptosis, proliferation, and cell signaling pathways [98], and are particularly relevant in tumors of neuroectodermal origin. Several ganglioside species, such as GM3, GD3, GD2, and GD1, have been reported to be upregulated in glioblastoma, neuroblastoma, melanoma, and BC, where they are associated with tumor progression and aggressive phenotypes [98,99,100]. In glioblastoma, GD3 has been identified as a predominant ganglioside species in tumor tissue by MS-based analyses, supporting its potential role as a molecular discriminator of tumor regions [99] (Table 3).

Additional studies have shown that variations in ganglioside composition, including fucosylated and acetylated derivatives, can distinguish tumor tissue from peritumoral or normal brain regions, reflecting intratumoral heterogeneity and invasive behavior [101,102,103,104,105].

In MS imaging techniques such as MALDI-MSI and DESI-MSI, the occurrence of frequently altered ganglioside species contributes to spatially resolved molecular patterns that support tissue classification and tumor delineation.

Other complex lipids, **glycerophospholipids (GPLs)** such as Phosphatidylcholine (PC), Phosphatidylethanolamine (PE), Phosphatidylinositol (PI), and Phosphatidylserine (PS) are widely reported to be upregulated in various tumor tissues relative to normal controls, consistent with increased membrane synthesis and remodeling in proliferating cells [106] (Table 3).

Alterations in glycerophospholipid composition have been consistently reported across multiple tumor types and represent some of the most robust molecular features detectable by MS in fresh tissue. In esophageal squamous cell carcinoma, increased accumulation of PC and PI, together with upregulation of enzymes involved in phospholipid remodeling, indicates enhanced membrane synthesis [107]. In colorectal cancer, spatially resolved MS analyses have shown differential distributions of lipid species: PC (37:5) is enriched in stromal and immune compartments, while PI (34:4) is more abundant in tumor cells [108]. Alterations in lysophospholipids have also been observed, including decreased levels of lysophosphatidylcholine (LPC) and lysophosphatidylethanolamine (LPE) in laryngeal and colorectal cancers, indicating disrupted phospholipid turnover. In advanced intrahepatic cholangiocarcinoma, increased FA 22:4 and GlcCer(d18:1/12:0), together with decreased lysophosphatidic acid (LPA) (16:0) and LPE (16:0), further highlight complex lipid remodeling and signaling alterations [94,109] (Table 3).

These changes are not typically interpreted as single biomarkers in intraoperative MS but rather as part of composite lipid signatures that enable discrimination between tumor and normal tissue and support margin assessment. These lipid patterns are among the most consistently exploited features in intraoperative MS platforms such as DESI-MS and REIMS.

Lipidomic-based methodologies are among the most widely used approaches for in vivo MS-based diagnosis in oncologic applications, as lipids are abundant, readily ionizable, and stable in fresh tissue. However, the molecular features exploited by intraoperative MS vary across platforms. Ambient techniques such as REIMS, MassSpec Pen, and SpiderMass predominantly rely on global lipid fingerprints, mainly composed of PC, PE, PI, and FAs, rather than individual biomarkers. In contrast, MS imaging approaches such as DESI-MSI and MALDI-MSI enable more detailed molecular characterization, including spatially resolved lipid distributions and the detection of selected metabolites, such as 2-hydroxyglutarate (2-HG), which enables identification of IDH-mutant gliomas (see Section 3.3). In addition, specific GSLs such as GD3 and GM3 have been detected in tumor tissues, particularly in brain tumors, contributing to tumor-associated molecular patterns in MS-based analyses.

The lipid changes described above are summarized in Table 3, highlighting the molecular patterns relevant for tumor characterization.

The continuous development of this field has increased the accuracy and sensitivity of detection, with useful implications for precise clinical diagnosis.

**Table 3:** Lipidomic signatures detected by intraoperative MS platforms.

Platform	Main Analyte Classes (Fresh Tissue)	Representative Lipid Patterns/Signatures	Clinical Applications
**REIMS (iKnife)**	Phospholipids (PC, PE, PI), FAs	Increased PC/PE/PI signals and FA-derived patterns in tumor tissue reflecting increased membrane synthesis and FA reprogramming [86,87,106]	Real-time tumor vs. normal discrimination; margin assessment; tissue classification [38,56,57,58,60]
**MassSpec Pen**	Phospholipids, FAs, metabolites	Altered phospholipid (PC, PE, PI) and FA composition associated with tumor metabolism, distinguishing malignant from normal tissue [86,88,89,90,91,106]	Intraoperative diagnosis; tumor classification; margin evaluation [50,63,64,65]
**SpiderMass**	Phospholipids, FAs	In vivo lipid profiles (PC and FAs alterations) reflecting tumor-associated metabolic changes [86,88,89,90,91,106]	Real-time tissue identification; intraoperative guidance [67,68,69,70]
**DESI-MS/DESI-MSI**	Phospholipids (PC, PE, PI), FAs, sphingolipids	Spatial lipid heterogeneity between tumor, stroma, and normal tissue: PC (37:5) enriched in stromal/immune regions; PI (34:4) enriched in tumor cells; altered LPC/LPE levels [94,106,107,108,109]	Margin assessment; tumor subtype classification; spatial tissue mapping [81,82]
**MALDI-MSI**	Phospholipids, sphingolipids (ceramides, gangliosides), FAs	Spatially resolved lipid and sphingolipid patterns; ceramide species (C16:0, C24:1); gangliosides (GD3, GM3, GD2) in tumor regions, PC/PI distributions [95,96,98,99,100,106,107]	Tumor delineation; histopathology correlation; spatial classification [30,52,54]
**PIRL-MS**	Phospholipids, FAs, metabolites	Combined lipid–metabolite signatures including PC and FA-derived signals supporting tumor classification [51,78,79,80,86,88,89,90,91]	Rapid intraoperative tumor classification; differentiation of tumor types [51,78,79,80]
**CUSA/SSI-MS**	Phospholipids (PC, PE), FAs, metabolites	PC, PE, and FA signals reflect tumor-specific composition in freshly aspirated surgical tumor tissue [41,72]	Rapid tissue characterization; intraoperative tumor identification, as support during tumor resection [41,72]
**Lipid changes commonly observed using MS platforms.**	FAs, GPL, sphingolipids	* ↑ long-chain FAs (e.g., palmitic acid (16:0), oleic acid (18:1)), ↑ MUFA/PUFA, ↑ desaturation index [86,87,90,91]; ↑ PC, PE, PI, including PC/PI remodeling [86,90,91]; altered ceramide/S1P balance, with changes in ceramide species (C16:0, C24:1) [95,96,97]; ganglioside upregulation in neuroectodermal tumors (e.g., GD3, GM3, GD2) [98,99,100]	Tumor vs. normal discrimination; margin assessment; subtype characterization

* ↑ indicates increased levels; ↓ indicates decreased levels.

### 3.3. Metabolomic Alterations

Metabolomic alterations in cancer arise from metabolic reprogramming driven by genetic changes, tumor microenvironment adaptation, and dysregulated signaling pathways. These changes involve a limited number of pathways that are consistently detectable by MS-based approaches, including enhanced glycolysis (e.g., increased lactate and pyruvate), altered tricarboxylic acid (TCA) cycle intermediates, changes in amino acid metabolism (e.g., glutamine, serine, and branched-chain amino acids—BCAAs), and the accumulation of selected oncometabolites such as 2-hydroxyglutarate (2-HG), fumarate, and succinate [33,110,111,112].

While a broad range of metabolites may be altered in tumors, only a subset is reliably detectable in fresh tissue and relevant for intraoperative MS. These include small metabolites (e.g., lactate and pyruvate), selected amino acid-related compounds, and other small endogenous metabolites (e.g., creatine, choline-related species, taurine), as well as oncometabolites such as 2-hydroxyglutarate (2-HG), together with pathway-related signatures that can be detected rapidly and contribute to tissue classification, tumor subtype discrimination, and mutation-specific profiling (Table 4).

Compared to lipid-based signatures, which currently dominate intraoperative MS workflows, metabolomic features provide additional biological and molecular context. Still, they are generally more sensitive to preanalytical factors such as ischemia and tissue handling. This limits their routine intraoperative use, although selected metabolites can still provide clinically relevant information in targeted contexts.

Recent developments in point-of-care MS have demonstrated the feasibility of rapid metabolomic profiling during surgery. For example, Wu et al. [109] developed a miniaturized, battery-powered linear ion trap mass spectrometer capable of analyzing fresh tumor biopsies within approximately 2 min. Using nanoESI-based sampling, the system detected a broad range of low-molecular-weight metabolites, with discrimination driven primarily by glycolysis- and energy metabolism-related signatures. This approach enabled differentiation between glioma and normal brain tissue, as well as IDH-mutant and wild-type tumors, highlighting the potential of metabolomics for rapid intraoperative classification and molecular stratification.

Current approaches increasingly aim to integrate lipid-based pattern recognition with targeted detection of selected metabolites, particularly oncometabolites suitable for rapid analysis. This combined strategy may support decisions during surgery by linking structural lipid changes with underlying metabolic pathways. In this context, MS-based techniques are emerging as valuable tools that complement conventional histopathology and support more precise tumor characterization during surgery.

The metabolite changes described above are summarized in Table 4, with emphasis on features that are detectable in fresh tissue and relevant for intraoperative MS applications.

**Table 4:** Metabolomic signatures detected by intraoperative MS platforms.

Platform	Main Analyte Classes	Representative Metabolites/ Signatures	Clinical Tasks
**REIMS (iKnife)**	Lipids (dominant), metabolites (minor)	Low-molecular-weight metabolites present in spectra, without specific metabolite biomarkers; classification driven by lipid-dominated profiles [38,56,57,58,60]	Tumor vs. normal discrimination; margin assessment [38,56,57,58,60]
**MassSpec Pen**	Lipids, metabolites	Small metabolites, including lactate, glutamate, ascorbate, choline-related species, creatine, and taurine, detected as part of combined lipid–metabolite profiles used for tissue classification [50,63,64,65]	Intraoperative diagnosis; tumor classification; margin evaluation [50,63,64,65]
**SpiderMass**	Lipids, metabolites	Low-molecular-weight metabolites and energy metabolism-related signals detected together with lipid profiles, contributing to global molecular patterns used for tissue classification [67,68,69,70]	Real-time tissue identification; intraoperative guidance [50,63,64,65]
**PIRL-MS**	Metabolites, lipids	Low-molecular-weight metabolites (mainly related to energy and amino acid metabolism) used for rapid tumor classification [78,79,80]	Rapid intraoperative tumor classification; tumor subtype discrimination [78,79,80]
**CUSA/SSI-MS**	Phospholipids (PC, PE), fatty acids, and metabolites	Low-molecular-weight metabolites detected in freshly aspirated tissue, without specific metabolite biomarkers; signals contribute to global lipid–metabolite profiles used for tissue classification [41,72,73]	Rapid tissue characterization; intraoperative tumor identification; support during tumor resection [41,72,73]
**DESI-MS/** **DESI-MSI**	Lipids (dominant), small metabolites	Small metabolites (e.g., lactate, TCA cycle-related intermediates) and oncometabolites such as 2-hydroxyglutarate (2-HG), detected in combination with lipid profiles for tumor classification and mutation-specific assessment for IDH-mutant gliomas [53,81,82]	Tumor vs. normal discrimination; tumor subtype classification; mutation status assessment (e.g., IDH); margin evaluation [53,81,82]
**MALDI-MS/MALDI-MSI**	Lipids, metabolites, peptides	Spatial distributions of small metabolites (e.g., lactate, nucleotide intermediates) and oncometabolites such as 2-hydroxyglutarate (2-HG), supporting tumor delineation and molecular stratification [30,52,54,81]	Tumor delineation; spatial tumor heterogeneity assessment; histopathology correlation; molecular stratification (e.g., IDH status) [30,52,54,81]
**Metabolic alterations commonly detected by MS platforms**	Small metabolites (central carbon metabolism, amino acid metabolism, oncometabolites)	Increased lactate and pyruvate; altered TCA cycle intermediates; elevated glutamine, serine, and BCAAs; oncometabolites such as 2-hydroxyglutarate (2-HG), fumarate, and succinate [33,109,110,111,112]	Tumor vs. normal discrimination; tumor subtype stratification; mutation-specific profiling

## 4. Clinical Applications in Oncology

Intraoperative MS has emerged as a powerful tool in oncologic surgery, enabling real-time molecular characterization of tissues directly within the operating room. By providing rapid and direct molecular profiling of metabolites and lipids, these techniques complement conventional histopathology and support real-time surgical guidance, particularly for tumor margin assessment and subtype discrimination. Among the most clinically advanced approaches are DESI, REIMS/iKnife, and handheld devices such as the MasSpec Pen, each offering distinct advantages in terms of speed, invasiveness, and tissue preservation. DESI-MS is widely used for ex vivo and near-real-time tissue analysis. Many studies have demonstrated the clinical feasibility of intraoperative MS in a variety of tumor types (CNS, breast, gynecological, urogenital, gastrointestinal, skin, head and neck), highlighting the growing role of ambient ionization MS in modern oncologic surgery (Table S1).

### 4.1. Brain Tumors

#### 4.1.1. DESI-MS for Intraoperative Diagnosis and Tumor Margin Assessment

DESI-MS has primarily been applied in ex vivo or near-real-time settings, using freshly excised or biopsy tissue samples rather than continuous in vivo analysis during surgery. Major advantages of DESI are rapid detection of tumor cells and the ability to properly assess tumor cell percentage in surgical margins—especially helpful in neurosurgical cases. DESI can distinguish healthy from pathological tissues with high sensitivity, specificity, and accuracy. Because surgical margins are not homogeneous, the average time to detect tumor cells is typically 1–2 min. Gentle spay systems allow multiple tissue scans without damaging the tissue. Gold standard pathology methods usually confirm results. The most recent innovation is the use of 3D DESI MS to analyze and characterize cancer [113].

DESI-MS was first introduced into intraoperative oncological surgery by Eberlin and colleagues in 2013 [39], as a molecular diagnostic tool in brain tumors (gliomas and meningiomas). Freshly excised or biopsy tissue from patients undergoing brain tumor surgery was pressed onto glass slides or analyzed directly under ambient conditions by directing charged solvent droplets onto the tissue surface, followed by mass-spectral acquisition over *m*/*z* ~100–1000 within seconds, capturing endogenous lipids and small metabolites with minimal fragmentation. The resulting lipidomic profiles, dominated by phosphatidylcholines (with the peaks corresponding to high-abundance PC 16:0/18:1 and PC 18:0/18:1 among the most discriminative features), phosphatidylethanolamines, and sphingolipids, allowed robust discrimination between glioma and normal brain tissue using multivariate statistical models. Notably, these lipid signatures not only enabled >90% accurate classification between glioma and normal tissue but also correlated with TCP present in histologic sections, and tumor grade, demonstrating that DESI-MS provides biologically meaningful information on tumor burden. This work established ambient lipidomic profiling as a powerful complement to frozen-section histopathology and laid the foundation for real-time molecular guidance during brain tumor surgery.

#### 4.1.2. DESI-MS for Molecular Characterization of Gliomas and Biomarker Detection

One major issue encountered in glioma surgery is the difficulty in reaching total gross resection, mostly due to the diffuse nature of this type of tumor. Among multiple mutations that occur in gliomas, isocitrate dehydrogenase (IDH) mutations are associated with better prognosis. IDH converts alpha-ketoglutaric acid (α-KG) into 2-hydroxyglutarate (2HG), which accumulates abnormally in cells. As noted above, these applications of DESI-MS in oncology are primarily based on the analysis of freshly excised tissue samples in a near-real-time setting, rather than continuous in vivo use. Shahi M et al. [114] demonstrated that DESI-MS can be used intraoperatively to assess IDH mutation status directly from glioma tissue samples. Using DESI-tandem MS, the authors detected elevated 2HG levels in both core tumor biopsies and margin samples, enabling rapid discrimination between IDH-mutant and wild-type tumors. In addition, the spatial distribution of 2HG provided valuable information on tumor infiltration at the margins, supporting surgical decisions regarding the extent of tumor resection. The timescale was suitable for deployment intraoperatively, highlighting the potential of DESI-MS as a real-time molecular analysis in glioma surgery. Pirro V et al. [76] further demonstrated the clinical utility of DESI-MS for intraoperative glioma assessment by detecting IDH mutations via 2HG as a robust marker and by rapidly (3 min) discriminating pathological tissue types using the obtained lipidomic profiles. High TCP at resection margins was obtained with high sensitivity and specificity for detecting infiltrative tumor tissue, thereby providing neurosurgeons with the molecular information necessary to guide the extent of tumor resection in real time. Diffuse glioma is the most common malignant brain tumor, with only curative treatment being surgical resection. Sometimes these surgical margins may still have percentages of cancer cells, and they account for tumor recurrence and malignant progression. Another study in this field, reported by Brown HM et al. [77], analyzed a large cohort of glioma specimens, including core tumor samples and margin biopsies, using DESI-MS to acquire lipidomic profiles directly from tissue without prior preparation. The authors used multivariate statistical models to distinguish IDH-mutant vs. IDH-wild type gliomas with high sensitivity and specificity, classify tumor versus non-tumor tissue, and estimate the percentage of infiltrating tumor cells at surgical margins. The workflow ran on a timescale compatible with an intraoperative diagnostic assay, demonstrating feasibility for real-time surgical guidance.

Several metabolites (e.g., gamma-aminobutyric acid (GABA), creatine, glutamic acid, carnitine, hexane-1,2,3,4,5,6-hexol (abbreviated as hexol), and N-acetylaspartate (NAA)) can be used to enhance diagnostic properties, with improved accuracy, sensitivity, and specificity when distinguishing normal brain tissue from glioma [115]. Oligodendrogliomas and astrocytomas can be rapidly and accurately diagnosed using lipidomic data acquired by ambient MS, with high cross-validation recognition rates (>97%), which aid subtype classification and grading [116]. Surgical decision support using DESI or MassSpec Pen data may help optimize resection and bypass the undesired systemic effects associated with chemotherapy and radiotherapy. Ambient MS enhances the detection of infiltrative tumor cells at resection margins compared with standard surgical resection and may improve patient outcomes [76,77,114,117].

#### 4.1.3. DESI-MS for Tissue Differentiation and Surgical Guidance

DESI-MS, either through positive or negative ion mode, can separate not only tumor vs. non-tumor tissue, but also gray vs. white matter (having distinct lipid compositions), which is critical in neurosurgery where tumor infiltration often occurs preferentially along white matter tracts, making visual and histological differentiation difficult, and a misclassification of white matter as tumor (or vice versa) can lead to incomplete resection. Different specificities and sensitivities are obtained in this context: a higher sensitivity (for detecting glioma-related lipid alterations, particularly PSs, PIs, and sulfatides) was observed when using negative ion mode, and a higher specificity (especially for discriminating tumor from gray matter based on phosphatidylcholines and sphingomyelins) when using positive ion mode. Combining the 2 methods of detection yields sensitivity and specificity of >90% across all 3 tissue classes (gray matter, white matter, glioma) [118].

Pirro et al. [119] showed that DESI-MS analysis of freshly excised tissue samples yields TCP estimates comparable to those obtained from frozen sections, supporting its use in assessing tumor infiltration. It also shortens analysis time by removing the need for freezing, cryosectioning, and additional imaging steps. In addition, it is a non-destructive technique that preserves tissue morphology for subsequent histopathological or molecular analyses, unlike other intraoperative diagnostic assays, which alter the sample.

Moreover, rapid molecular data from DESI, together with 3D MRI mapping, further enhances clinical decision-making during brain tumor surgery [120]. Similar performances were observed when comparing gliomas with meningiomas and astrocytomas in terms of the rapidity of diagnosis and near-real-time tumor margin assessment aided by neuronavigation systems [46]. Complementarily, Jarmusch et al. [121] developed multivariate discriminant models based on combined lipid and metabolite profiles, enabling accurate classification of multiple brain tumor types, gliomas, meningiomas, and pituitary tumors, and highlighting the importance of rapid detection and diagnosis in all these variants.

GBMs are highly aggressive and heterogeneous tumors, a characteristic that significantly contributes to their resistance to chemotherapy and radiotherapy. Increasing evidence indicates that lipid metabolism plays a central role in this intratumoral heterogeneity, influencing tumor viability and therapeutic response. Henderson et al. [122] applied 3D DESI-MS lipid imaging in a GBM xenograft model and demonstrated that this approach reveals a much broader and more complex lipid heterogeneity than conventional hematoxylin and eosin (H&E) staining alone, enabling clear molecular differentiation between hypoxic and viable tumor regions. This lipid-based molecular mapping improved the delimitation of biologically distinct tumor regions. It offered insights into metabolic adaptations associated with hypoxia and aggressiveness, thereby highlighting the added value of DESI-MS lipid imaging for uncovering GBM heterogeneity and advancing understanding of mechanisms of treatment resistance.

#### 4.1.4. REIMS, PIRL, and CUSA/SSI-MS for Intraoperative Brain Tumor Analysis

Although DESI-MS shows strong diagnostic potential, its in vivo intraoperative use remains technically challenging. To address this, REIMS-based approaches have been developed, enabling real-time analysis of lipid profiles directly from surgical aerosol generated during electrosurgery, without prior tissue preparation. The iKnife, which couples an electrosurgical knife to a mass spectrometer, allows rapid molecular profiling of the generated aerosol within seconds. However, this method has important limitations, as it is destructive, restricted to electrosurgical procedures, and relies on indirect correlation with histopathology, as intact tissue is not preserved [123].

Using this approach, low- and high-grade glioma characterization, grade II and III astrocytomas, and normal brain tissue could all be distinguished with high accuracy, demonstrating the clinical value of rapid lipidomic feedback for intraoperative diagnosis. Van Hese et al. [59] showed that REIMS-guided glioma surgery enabled real-time tissue classification with histological diagnoses confirming the intraoperative MS findings with 100% accuracy. Balog et al. [38] highlighted the broad applicability of REIMS, revealing distinct MS patterns that reflect lipidomic diversity across different histological subtypes and between primary and metastatic tumors.

The major drawback of REIMS—the extensive tissue damage induced by cautery—can be mitigated by using a different ambient MS tool, PIRL MS. PIRL delivers ultrafast laser pulses that ablate tissue more gently than electrocautery, preserving tissue structure while enabling rapid molecular analysis. This approach has been applied in medulloblastoma surgery, the most prevalent malignant pediatric brain tumor. Woolman et al. [78,124] demonstrated that PIRL-MS can rapidly detect within 5–10 s and robustly identify the four medulloblastoma subgroups (WNT, SHH, Group 3, Group 4) and molecular biomarkers crucial for accurate classification. Failed classifications after surgical resection may be due to tissue damage. Thus, PIRL-based analysis reduces tissue damage compared with REIMS, thereby improving the reliability of intraoperative subgrouping of tumor samples and supporting personalized surgical treatment.

Better images of cancer tissues were obtained when combining the rapidity of PIRL with the software interface of a DESI MS module. This dual approach provides high-quality, robust molecular images that are consistent with other ambient ionization methods and are fast enough for intraoperative decision-making. Katz et al. [125] demonstrated that combining PIRL with a DESI module enables clear tissue visualization and lipidomic mapping in seconds. In addition, Woolman et al. [79] achieved 10 s classification of pediatric brain tumors, differentiating medulloblastomas from pilocytic astrocytomas and ependymomas, and molecular subgrouping. Similarly, Fiorante et al. [80] applied PIRL-MS to identify, in 10 s, different spinal tumors, ranging from meningiomas to schwannomas, as well as intradural extramedullary spinal neoplasms such as myxopapillary ependymoma, neurofibroma, paraganglioma, and solitary fibrous tumor. The quality of the results was similar to that of genomic, transcriptomic, or methylomic assays, but at orders of magnitude faster, highlighting the potential of PIRL-based ambient MS for rapid, intraoperative molecular classification of a wide variety of central nervous system (CNS) tumors.

Schäfer et al. [41] introduced CUSA/SSI-MS as an MS-based approach for near-real-time tissue identification in a surgical context by coupling ultrasonic surgical aspiration with sonic spray ionization. The method was evaluated on ex vivo and post-mortem human brain samples, including tumors (mainly gliomas) and non-tumorous brain tissue, without additional sample preparation. Distinct lipidomic profiles were observed between tumor and non-tumor tissues, primarily driven by differences in phospholipids and other lipid species. Classification using PCA/LDA models enabled rapid tissue identification, although performance metrics were derived from a limited dataset and not reported as standard clinical measures (e.g., sensitivity or specificity). As the CUSA procedure is already widely used in neurosurgical workflows, the obtained data support the feasibility of CUSA/SSI-MS for intraoperative tissue identification and margin assessment in brain tumor surgery.

### 4.2. Breast Tumors

#### 4.2.1. Clinical Challenges and Role of Intraoperative MS in BC Surgery

Invasive ductal cell carcinoma (IDC) represents the most frequent histological type of malignant BC, accounting for approximately 70% of all BC.

Contemporary surgical management favors primarily breast-conserving surgery (BCS) over radical mastectomy; however, achieving tumor-free resection margins while preserving healthy glandular tissue remains a major intraoperative challenge. A major challenge is represented by the molecular heterogeneity of BC subtypes, which differ in prognosis and therapeutic response and therefore require accurate identification during surgery. Incomplete margin assessment remains a leading cause of re-excision in BCS, underscoring the need for rapid, reliable intraoperative diagnostic tools [60,126].

In this context, different types of MS-based techniques have emerged as powerful tools for rapid tissue characterization, proper diagnosis of cancer tissue, delineation from normal tissue, establishment of resection margins, and rapid sampling and results. Neagu et al. [60], using multiple MS-based proteomics approaches, have identified dysregulated protein networks and candidate biomarkers in IDC to improve diagnosis and treatment, as preserving glandular tissue during breast-sparing surgery and achieving cancer-free resection margins are very difficult. For intraoperative use, ambient MS technologies, including the MasSpec Pen and REIMS/iKnife, show particular promise by enabling rapid discrimination between cancerous and normal tissue and by assisting margin assessment [60]. Iknife is fast and provides real-time margin information with high sensitivity and specificity, but it is destructive, time-consuming, electrosurgery-dependent, and generates complex datasets that can limit large-scale training. Currently, AI-enhanced learning approaches have been integrated with iKnife data, as demonstrated by Santilli et al. [127], who reported high sensitivity, specificity, and accuracy in discriminating between tumor and normal breast tissue and between tumor and normal skin tissue.

#### 4.2.2. REIMS/iKnife and PIRL-MS for Real-Time Intraoperative Tissue Analysis

Certain breast regions require longer analytical times, particularly adipose-rich areas, which are highly abundant in lipids and can complicate molecular interpretation. A way to mitigate this issue is to use PIRL MS, which enables faster tissue ablation and shorter detection time. However, breast fat contains lipids, which can still interfere with the results. In this context, polarimetric imaging can robustly guide PIRL-MS toward regions with lower fat content to avoid signal contamination and shorten overall MS analysis time. Intraoperative tumor heterogeneity, such as variations in vasculature, stromal composition, and resection margins, plays a critical role in patient outcomes [128]. The tumor-associated stroma undergoes molecular alterations in BC and represents an important diagnostic target. Hence, REIMS has been applied for the rapid analysis of surgical vapors, with complementary support from DESI MS for the analysis of breast stroma in breast-conserving strategies [129]. Using the iKnife, BC tissue has been identified with high sensitivity and specificity, with mean data acquisition and analysis times of less than 2 s, highlighting its suitability for real-time intraoperative guidance [56].

#### 4.2.3. Tumor Heterogeneity and Multimodal Intraoperative Assessment

Tata A et al. [130] used combined contrast-enhanced MRI and MS imaging to characterize this heterogeneity, in which MRI localized tumor regions using gadoteridol, and DESI-MS provided complementary molecular characterization.

BC necrosis is a poor prognostic marker, commonly associated with aggressive tumors and unfavorable outcomes. Necrotic regions exhibit altered optical depolarization properties and present distinct molecular signatures, making them attractive diagnostic targets. DESI MS rapidly detects necrosis-specific ions directly from tissue, avoiding tissue sectioning and thereby accelerating identification and enabling near-real-time intraoperative assessment [131]. Conventional DESI MS tissue preparation can be time-consuming. Using tissue smears instead of snap-freezing and cryosectioning the samples has resulted in a significant improvement in intraoperative analysis and detection. However, smears result in lower ion concentrations and fewer cancer biomarkers. Although smears yield lower ion abundances and reduced biomarker intensities, comparative analyses show no statistically significant loss in discriminatory power between smears and tissue sections, indicating their utility for rapid cancer profiling [132].

#### 4.2.4. DESI-MS and MasSpec Pen for Molecular Profiling and Clinical Applications

The developed handheld device, the MasSpec Pen, was used for minimally invasive, nondestructive analysis of BC for rapid detection. It enables rapid molecular profiling with minimal tissue damage and has identified multiple diagnostically relevant lipid biomarkers, including phosphatidylinositols (*m*/*z* 885.550, identified as PI(38:4), *m*/*z* 863.565, identified as PI(36:1)), phosphatidylglycerols (*m*/*z* 773.542, identified as PG(36:2)), and several FA such as *m*/*z* 303.233, identified as FA(20:4), and *m*/*z* 281.249, identified as FA(18:1), demonstrating strong potential for real-time surgical control [50].

DESI-MS analysis of tissue sections has demonstrated that tumor heterogeneity is closely related to the spatial distribution of specific molecular signals. A marked abundance of cancer-associated lipid biomarkers is predominantly localized in the cytoplasm of cancer cells. In contrast, heterogeneous components such as stroma and vasculature often show weaker or less consistent correlations with biomarker abundance. Subtle intratumoral heterogeneities that may go unnoticed by conventional histopathology are more readily detected with MS-based imaging approaches [133]. In addition, molecular drivers of tumor aggressiveness, including Human Epidermal Growth Factor Receptor 2 (HER2) overexpression and tumor protein p53 (p53) mutations, are strongly associated with disease progression and metastatic potential. DESI MS can help identify lipid markers associated with specific phenotypic and genotypic profiles, enabling molecular stratification of BC subtypes [134]. The applicability of DESI-MS has also been demonstrated by Zhang et al. [135] for the accurate detection of metastatic BC. Moreover, DESI-MS has been employed to monitor therapy-induced molecular changes, by studies targeting polo-like kinase 1 (PLK1), a key regulator of cell cycle progression and carcinogenesis, which was used either as a biomarker or as a target for specific therapies.

Alterations in surface lipid markers before and after treatment with a PLK1-silencing chimeric aptamer (EpDT3-siPLK1, which downregulates PLK1) were detected using DESI-MS by Jayashree B et al. [136], thus offering a powerful platform for evaluating therapeutic response at the molecular level.

### 4.3. Digestive System Neoplasms

These tumors are highly diverse and share common clinical challenges, including the need for rapid intraoperative detection, accurate margin delineation, and reliable biomarker identification for prognostic and therapeutic purposes.

#### 4.3.1. Colorectal Cancer

DESI-MS was used to reveal heterogeneity in colorectal adenocarcinoma that is not discernible with conventional analytical approaches. The 3D-DESI-MS dataset acquired from human colorectal adenocarcinoma biopsies was projected onto a two-dimensional manifold to detect metabolic clusters that are not visible with linear analytical methods [137]. Furthermore, DESI-MS has demonstrated the ability to distinguish histological variants of colorectal adenocarcinoma and their corresponding liver metastases, revealing complex lipidomic profiles dominated by PI, PE, PS, plasmalogens, phosphatidic acids, phosphatidylglycerols, ceramides, sphingolipids, and sulfatides [138].

Rapid detection was achieved using REIMS-based iKnife methodology for colorectal cancer and colonic adenomas, enabling accurate tissue classification and identification of cases associated with poor prognosis. This approach achieved high diagnostic accuracy while effectively differentiating histological features during endoscopic procedures [58].

#### 4.3.2. Esophageal Cancer

In esophageal cancer, lipidomic profiling using both Ultra-Performance Liquid Chromatography–Electrospray Ionization–Mass Spectrometry (UPLC–ESI-MS) and DESI-MS yielded concordant diagnostic results, with observed differences limited primarily to ion adduct formation across acquisition modes [139].

#### 4.3.3. Pancreatic Cancer

In pancreatic cancer surgery, where precise tumor margin assessment is critical to treatment success, conventional cold tissue preparation is often unfeasible intraoperatively, so the MasSpec Pen has been introduced as a rapid, nondestructive alternative. By detecting alterations in lipid and metabolite profiles in vivo and ex vivo, this technology enables accurate diagnosis of pancreatic ductal adenocarcinoma with high predictive performance [65].

### 4.4. Urogenital Tumors

DESI MS was used with promising results to diagnose urogenital cancers. It provides valuable information on disease state and tumor grade based on lipid profiles in tissue sections [140].

#### 4.4.1. Bladder Cancer and Transitional Cell Carcinoma

Bladder cancer was distinguished from normal tissue with high accuracy by analyzing GPL mass spectral profiles, with performance comparable to conventional H&E histopathology [141]. Invasive transitional cell carcinoma of the urinary bladder has also been successfully detected using DESI MS, where the spatial distributions of particular GPL, sphingolipids, and FFA in both the negative and positive ion modes were compared to serial H&E-stained sections [142].

#### 4.4.2. Renal Tumors: Renal Carcinoma vs. Oncocytoma Differentiation

Renal carcinoma (RCC) can be difficult to distinguish from renal oncocytoma (the most common benign renal tumor), as it can closely mimic malignant subtypes. DESI MS showed 100% predictive value for differentiating oncocytoma from RCC, whereas conventional histological methods are very unreliable, especially between chromophobe RCC and oncocytoma [143]. Additionally, DESI-MS enabled differentiation among papillary RCC, clear cell RCC, and normal renal tissue, primarily through GPL detection in negative ion mode, with misclassification rates below 20% compared with the validation set [144].

#### 4.4.3. Prostate Cancer

DESI-MS has also been applied to prostate cancer, where cholesterol sulfate was detected almost exclusively in cancerous tissue, including precancerous lesions [145]. Rapid molecular assessment is particularly valuable in this setting. Banerjee et al. [146] showed that DESI-MS imaging of small metabolites, carbohydrates, and lipids revealed distinct metabolic alterations in malignant prostate cells compared with benign tissue, and the diagnostic information was obtained within ≈1 min, considerably faster than conventional histopathological examination, which typically requires around 20 min.

### 4.5. Skin Tumors

#### 4.5.1. REIMS/iKnife in Skin Cancer Surgery

REIMS-based iKnife technology has also been applied for the surgical management of basal cell carcinoma (BCC). A critical consideration for REIMS is its reliance on developing reliable deep learning models capable of accurately classifying tissues based on their metabolomic signatures. In this context, Fooladgar et al. [61] demonstrated the feasibility of iKnife-guided margin detection during BCC resection, identifying the tumor margins with high accuracy and sensitivity. This study was the first to incorporate uncertainty estimation into REIMS-based surgical evaluation.

#### 4.5.2. DESI-MS in Squamous Cell Carcinoma

Complementary ambient MS approaches have been used in other epithelial malignancies. Thus, DESI-MS has proven effective in differentiating oral tongue squamous cell carcinoma (SCC) from adjacent normal epithelium, with reported accuracy rates of 95% for SCC vs. normal tissue and 93% when discriminating SCC, adjacent normal, and normal epithelium, emphasizing its diagnostic reliability in head and neck oncology [147].

In addition to tumor detection, DESI-MS has been used to provide relevant prognostic information, such as the tumor stroma ratio (TSR). TSR is a documented prognostic marker, with stroma-rich tumors associated with poorer outcomes and increased risk of recurrence. Woolman et al. [148] demonstrated that DESI-MS can rapidly estimate TSR in SCC by identifying specific lipid biomarkers, including *m*/*z* 773.53 [PG(18:1/18:1)–H]^−^, *m*/*z* 835.53 [PI(34:1)–H]^−^, and *m*/*z* 863.56 [PI(18:1/18:0)–H]^−^.

### 4.6. Gynecological Tumors

#### 4.6.1. Ovarian Cancer: Intraoperative Diagnosis Using MasSpec Pen and DESI-MS Imaging

Accurate intraoperative tissue diagnosis during ovarian cancer (OC) surgery is critical to maximize tumor excision and define therapeutic options. Using the MasSpec Pen for OC analysis, Sans et al. [63] demonstrated rapid molecular extraction and real-time tissue diagnosis, achieving reliable discrimination between OC and adjacent fallopian tube or peritoneal tissues. Particular MS profiles were observed in normal tissue, low-grade, and high-grade serous ovarian cancer, highlighting that the assay is sensitive to disease-state-specific molecular alterations.

Ovarian high-grade serous carcinoma (HGSC) results in the highest mortality among gynecological cancers, due to its aggressive biology and rapid progression, whereas serous borderline ovarian tumors (BOTs) exhibit more indolent behavior but retain the potential to progress to low-grade serous carcinoma. DESI-MS imaging has enabled clear visualization of characteristic histopathological features, including papillary branches in serous BOT, and allowed for characterization of tumor heterogeneity, such as adjacent necrosis and reactive stroma in HGSC. DESI-MS-derived metabolic signatures have been shown to correlate with tumor aggressiveness, enabling the identification of predictive markers with potential prognostic value [149]. Moreover, rapid discrimination between HGSC and normal ovarian stroma has been achieved using the MasSpec Pen [50].

#### 4.6.2. Ovarian Cancer: Real-Time Tissue Analysis Using REIMS/iKnife and SpiderMass

In addition to DESI-MS and Mass Spectrometry Pen approaches, REIMS-based iKnife technology has been used in gynecological surgery by analyzing MS lipidomic profiles from diathermy-generated aerosols. Using this approach, BOTs were readily distinguishable from invasive OC, with strong concordance between iKnife-based classifications and conventional histopathological assessment [57].

Fatou et al. [40] used another MS-based surgical tool, the SpiderMass, coupled to a 3D ion trap mass spectrometer (with the ion source removed) during gynecological surgery for differentiating high-grade serous ovarian carcinoma from benign ovarian tissue, based on lipidomic profiles containing mainly GPLs, FAs, and diacylglycerols (DG) within the *m*/*z* 200–1000 range. The study showed that, although the analysis was performed ex vivo on ovarian tissue biopsies, SpiderMass generates and transfers ions to the MS on millisecond timescales during laser ablation. The data were collected for ~30 s continuously and then averaged to produce representative spectra for comparison between tumor and normal tissue, enabling real-time detection with minimal tissue damage. Subsequent studies using SpiderMass [67] have focused on methodological optimization and in vivo validation in animal models.

### 4.7. Endocrine Tumors

#### 4.7.1. Thyroid Tumors: MasSpec Pen for Tumor Classification

The MasSpec Pen demonstrated very high sensitivity, specificity, and accuracy (all above 90%) in differentiating papillary thyroid carcinoma (PTC) from follicular thyroid adenoma (FTA) by generating rich molecular profiles comprising metabolites, lipids, and proteins. Importantly, MasSpec Pen analysis causes no observable tissue damage or stress, supporting its suitability for in vivo cancer diagnosis [50].

#### 4.7.2. Thyroid Tumors: DESI-MS for Molecular Profiling and Tumor Differentiation

In addition to primary tumor identification, lymph node metastases from thyroid cancer have been successfully detected using DESI-MS [142]. Also using DESI, molecular signatures of normal thyroid tissue, benign follicular adenoma (FTA), malignant follicular carcinoma (FTC), and papillary thyroid carcinoma (PTC) were compared and used for differentiation. This approach shows particular promise for improving the diagnostic capabilities of fine-needle aspiration biopsies, which remain inconclusive in up to 20% of cases, potentially delaying or missing cancer diagnoses. Another study [150] demonstrated that MS-based metabolic profiling can accurately classify thyroid nodules using fine-needle aspiration samples, highlighting the potential of MS to provide rapid, molecularly informed diagnostic information. However, based on preoperative samples, these findings support the feasibility of translating similar approaches to intraoperative applications.

A particular subgroup of thyroid tumors—oncocytic tumors—is characterized by an eosinophilic, granular cytoplasm due to mitochondrial accumulation. Using DESI-MS, Zhang et al. [151] demonstrated that oncocytic tumors exhibit a high abundance and chemical diversity of cardiolipins (CL), including numerous oxidized species, reflecting mitochondrial dysfunction. In addition to cardiolipins, altered levels of other GPL, FFA, and metabolites were observed, enabling discrimination between oncocytic, non-oncocytic, and normal thyroid tissues.

All these applications highlight the potential of MS to provide rapid molecular guidance during surgery, complementing or even surpassing histopathological techniques. REIMS/iKnife is currently the most clinically used intraoperative MS method for real-time surgical guidance. At the same time, DESI-MS is the most widely used ambient ionization MS overall, being primarily applied ex vivo or in situ (for tissue imaging and research), rather than continuously in vivo during surgery. MasSpec Pen is a rapidly emerging clinical technology with strong performance, but it has been reported less frequently in actual clinical use than REIMS and DESI so far, mainly because of its more recent development and ongoing validation.

## 5. Computational Approaches for Intraoperative Mass Spectrometry

Intraoperative MS generates highly complex, high-dimensional datasets that reflect the molecular composition of tissues in real time. To translate the complexity of MS spectra into useful information for surgical decision-making, robust computational methods capable of reducing data complexity and accurately classifying tissue types are required.

### Evidence Quality and Validation in Intraoperative MS Studies

When evaluating the performance of computational models applied to intraoperative MS data, reported metrics such as sensitivity, specificity, accuracy, and concordance should be interpreted with caution, as they are derived from highly heterogeneous study designs. Across the literature, datasets may include ex vivo banked tissues, freshly excised intraoperative specimens, biopsy samples, tissue smears, frozen sections, margin samples, or surgical aerosols/aspirates, each with different preanalytical constraints and sources of variability.

In addition, validation strategies differ substantially between studies. Some investigations report only internal cross-validation, whereas others include independent test sets or, more rarely, external validation cohorts. The level at which data are split is also critical. Performance estimates based on spectrum-level splitting may overestimate diagnostic performance when spectra from the same patient are represented in both training and test datasets. Patient-level validation is more clinically meaningful, especially for studies aimed at supporting intraoperative surgical guidance.

Another important source of heterogeneity is the definition of ground truth. In most studies, histopathology serves as the reference standard, but some also incorporate immunohistochemistry, TCP estimation, or molecular testing such as IDH mutation status. These differences affect the interpretation and comparability of reported results, particularly when molecular classification and tissue classification are discussed together.

For these reasons, direct comparison of performance metrics across intraoperative MS studies should be avoided unless the validation framework, specimen type, and reference standard are clearly aligned. Whenever available, patient-level results, cohort size, and confidence intervals provide a more realistic estimate of clinical performance than spectrum-level classification alone. Future studies would benefit from more standardized reporting of training and test cohorts, explicit separation of internal and external validation, patient-level performance metrics, and clearer descriptions of the pathological or molecular reference standard used.

Among the computational approaches, Principal Component Analysis (PCA) is commonly used as an initial step for dimensionality reduction. By transforming large spectral datasets (spectra with thousands of *m*/*z* points) into a smaller number of “principal components”, PCA facilitates data visualization, highlights underlying patterns, and supports the identification of distinct tissue groups, such as tumor and non-tumor regions.

Building on this, Linear Discriminant Analysis (LDA) is often applied as a supervised classification method, used to maximize separation between predefined classes, enhancing the distinction between healthy and cancerous tissues and facilitating rapid, interpretable classification during surgery. Its main advantages are prediction and classification based on already-labeled spectra; e.g., after training the model on spectra from glial tumors and healthy tissue, LDA can classify a new spectrum in real time into the correct category.

Support Vector Machine (SVM) is another widely used classification approach, particularly suitable for handling complex and high-dimensional MS data. It enables a reliable classification, sometimes more powerful than LDA in non-linear situations, differentiating, for example, between multiple types of brain tumors (astrocytoma, oligodendroglioma, metastasis) based on metabolomic signatures.

These methods have been applied to MS data across various tumor types. Fournier et al. [152] discuss DESI-MS conceptual and technical advances for diagnosing tumor margins during surgery, highlighting the use of multivariate statistical methods (including PCA for exploratory analysis of complex MS datasets) to differentiate healthy vs. tumor tissues and subtype tissues based on lipid signatures. D’Hue et al. [147] demonstrated that PCA combined with LDA of DESI-MS spectra can distinguish squamous cell carcinoma from normal epithelium, a feature relevant to intraoperative tumor margin assessment.

Balog et al. [38] applied PCA for feature extraction from intraoperatively collected mass spectra using REIMS (iKnife), then used machine learning (e.g., PCA and SVM, PCA and LDA) to classify tissue types during live surgery. PCA here reduces the dimensionality of high-dimensional surgical MS data, enabling robust real-time tissue differentiation when combined with other approaches. Other researchers [153] applied PCA-LDA to REIMS metabolic data to compare MS classification accuracy vs. histopathology for oral cavity cancer resections, clearly linking PCA with surgical MS imaging (MSI).

Balog et al. [38] in their foundational study applied REIMS coupled with iKnife for real-time tissue classification, and multivariate analysis, including PCA and LDA, was used to separate cancerous and non-cancerous spectra and to build classification models for intraoperative use. They demonstrated that LDA could be used with MS for real-time surgical tissue identification. The study reports high classification accuracy (>97% in the reported study, based on cross-validation) for tissue type discrimination t using PCA and LDA on MS profiles obtained from electrosurgical vapor, including in vivo and ex vivo samples, supporting the feasibility for real-time MS-guided diagnostics during surgery. However, reported performance metrics in intraoperative MS studies are highly dependent on dataset construction and validation strategy. In particular, performance may be overestimated when spectra from the same patient are included in both training and test sets or when only internal cross-validation is used. Van Hese et al. [59] developed REIMS classification models that used multivariate statistical analysis (PCA and LDA) to distinguish between different brain tissues (normal vs. glioma subtypes) in a surgical context. Their paper shows classification of multiple high- and low-grade glioma tissue types using LDA models applied to MS spectra in neurosurgery.

Phelps et al. [57] used REIMS and the iKnife approach in a clinical study on gynecologic surgical specimens. Multivariate analysis, including PCA and LDA, was performed to classify normal, borderline, and malignant tissue intraoperatively. LDA, together with PCA, was part of the statistical workflow used to separate multiple tissue classes with high reported sensitivity and specificity depending on the validation approach in surgical gynecology. Barber et al. [154] summarized REIMS surgical applications and reported numerous studies in which PCA-LDA classification models were applied to intraoperative MS data for tissue discrimination. PCA-LDA has been commonly used in REIMS research to classify normal vs. pathological tissue in surgical contexts.

Giordano et al. [25] further studied cancer resection and how to better use MS techniques in this context. Probe electrospray ionization mass spectrometry (PESI-MS) was used to analyze biopsy tissues, and an SVM algorithm was used to classify mass spectra as tumor vs. non-tumor during surgical evaluation, distinguishing liver cancer tissues (HCC) from healthy tissues in a large Italian population group, to guide cancer resection. This study aimed to confirm that the instrument’s classification performance was not influenced by genetic background, disease status, etiology, or operating room protocols. To this end, we evaluated the suitability of an HCC dataset developed for this system. The HCC dataset, comprising 200 real specimens from Japanese patients, was analyzed, and its ability to correctly distinguish tumor from non-tumor tissues in Italian patients was assessed. The Japanese database was then expanded with Italian samples, leading to improved model performance, as reflected in clearer separation of samples in the SVM score distribution and higher diagnostic accuracy compared with histopathological assessment.

In another study [155], the same team compared the PESI-MS data obtained from HCC and mass-forming cholangiocarcinoma with non-tumor liver tissue and classified the findings using SVM. While not explicitly described as “intraoperative,” the workflow was designed for rapid, point-of-care use and demonstrated >94% diagnostic accuracy reported in the study cohort, depending on dataset composition and validation strategy, making it highly relevant for near-intraoperative margin assessment and surgical decision support.

Huang et al. [156] analyzed PESI-MS spectra from papillary thyroid carcinoma and adjacent tissues. They classified them using multiple machine learning algorithms, including SVM, achieving high classification performance for tumor vs. normal and lymph node metastasis status. Although this study includes preoperative data, it demonstrates that the association of PESI-MS with SVM models enables rapid diagnosis, potentially relevant for intraoperative settings, where quick and reliable classification is critical for determining resection extent.

While PCA-, LDA-, and SVM-based models have shown strong performance in research and early clinical studies, their use in real-time intraoperative applications remains challenging due to several practical constraints. One of the main considerations is the time required for data processing, as clinical decisions during surgery rely on results to be available within a clinically meaningful timeframe (usually within seconds). In practice, overall latency depends not only on the classification algorithm itself, but also on spectral acquisition and preprocessing steps.

From a technical perspective, most current implementations rely on workstation-level systems integrated with the mass spectrometer, and translating them to more compact or portable solutions is still under development. Emerging approaches based on edge computing and embedded machine learning aim to enable on-device, low-latency inference directly integrated with MS platforms, which is particularly relevant for point-of-care and intraoperative applications. However, these solutions are still in early stages of development and require further validation before routine clinical implementation. In addition, the use of such models in clinical settings raises regulatory considerations, as software-based decision support systems may be subject to medical device regulations and require transparency, reliability, and continuous performance monitoring. Careful validation is also essential, including reproducibility across centers and stability over time. Variations in instrumentation, acquisition protocols, or patient populations may affect model performance, making periodic updating and validation necessary.

Finally, interpretability remains important, as clinical decisions depend on models whose results can be clearly understood and trusted by the surgical team.

Addressing these aspects, together with the availability of large, well-annotated datasets and improved integration into surgical workflows, will be important for the broader clinical adoption of computational MS approaches.

The combination of dimensionality reduction and classification techniques represents a critical step toward transforming complex MS data into clinically relevant information, supporting more precise and informed decisions in oncological surgery.

Further progress in this field will depend on the availability of large, well-annotated datasets and on validation through multicenter, prospective intraoperative studies to assess the robustness and generalizability of these approaches across different clinical applications.

## 6. Conclusions and Future Perspectives

Intraoperative MS has emerged as a promising approach in oncologic surgery, enabling real-time tumor detection and supporting more accurate resections. A range of technologies, including REIMS, the MasSpec Pen, PIRL-MS, SpiderMass, CUSA/SSI-MS, and DESI-based approaches, have demonstrated the capacity to distinguish malignant from normal tissue and to provide molecular information that complements conventional histopathology. Among these, REIMS is currently the most clinically developed online intraoperative MS technique, supported by multicenter studies demonstrating real-time discrimination between tumor and normal tissue in breast, ovarian, colorectal, and brain cancers. The MasSpec Pen has shown high diagnostic accuracy in prospective intraoperative studies, particularly in breast and thyroid surgery. At the same time, PIRL-MS has proven especially valuable in sensitive applications such as brain tumor surgery, where rapid molecular classification can be achieved with minimal tissue damage. Similarly, SpiderMass has shown promising results in early translational studies, particularly in head and neck oncology, enabling minimally invasive real-time tissue analysis. Emerging techniques, including CUSA/SSI-MS, which provides real-time brain tumor characterization in neurosurgery and inline DESI-MS for near-real-time tissue analysis, further expand the range of applications for intraoperative MS across different surgical contexts. Despite these advances, intraoperative MS remains largely translational rather than fully integrated into routine clinical practice, with current limitations related to technical variability, challenges in workflow integration (including instrument size, which may range from portable handheld devices to mass spectrometers exceeding 100–200 kg, power requirements typically in the range of several hundred watts to kilowatts depending on the platform, high acquisition and maintenance costs, and compatibility with sterile environments), and the need for consistent interpretation of complex spectral data. Overall, while the potential of MS-guided surgery is clear, further validation and refinement are required before it can become a standard component of oncologic procedures.

Future progress in intraoperative MS will depend on several key developments. Large-scale, multicenter clinical studies are needed to confirm diagnostic performance across different tumor types and surgical settings. At the same time, standardizing sampling protocols, spectral libraries, and decision thresholds will be essential to ensure reproducibility and facilitate regulatory approval. Improvements in instrumentation and data analysis will be essential for moving these approaches closer to routine clinical use, supported by ongoing efforts toward miniaturization, improved portability, and better integration of MS systems into the operating room environment. Studies exploring battery-operated Orbitrap systems [157] illustrate that technical adaptations are feasible, but broader clinical adoption will require further refinement and operational validation. In parallel, advances in machine learning and other computational approaches have the potential to improve the speed and reliability of tissue classification, particularly when dealing with complex molecular tumor-related data. Thus, intraoperative MS has the potential to transform oncologic surgery by providing molecular insights that extend beyond conventional histopathological assessment. Continued interdisciplinary research, technological refinement, and rigorous clinical validation will be essential to advancing MS-guided surgery toward routine oncologic practice and improving patient outcomes in oncology.

## Data Availability

No new data were created or analyzed in this study. Data sharing does not apply to this article.

## Supplementary Materials

The following supporting information can be downloaded at: https://www.mdpi.com/article/10.3390/molecules31081287/s1, Table S1. Clinical implementation of intraoperative MS techniques based on reported studies.

## Abbreviations

The following abbreviations are used in this manuscript:

2HG	2-hydroxyglutarate
5-ALA	5-aminolevulinic acid
AIMS	ambient ionization mass spectrometry
APCI	atmospheric pressure chemical ionization
API	atmospheric pressure interface
BCAAs	branched-chain amino acids
BC	breast cancer
BCS	breast-conserving surgery
ccRCC	clear cell renal cell carcinoma
CLs	cardiolipins
CNS	central nervous system
CRC	colorectal cancer
CT	computed tomography
CUSA/SSI-MS	Cavitron Ultrasonic Surgical Aspirator/Sonic Spray Ionization
DESI-MS	Desorption Electrospray Ionization—MS
EOC	epithelial ovarian cancer
ESI	Electrospray ionization
FAs	fatty acids
FFAs	free fatty acids
FTA	follicular thyroid adenoma
FTC	follicular thyroid carcinoma
GBM	glioblastoma multiform
GC MS	Gas chromatography MS
GDs	diacylglycerols
GPLs	glycerophospholipids
GSLs	glycosphingolipids
H&E	Hematoxylin & Eosin
HCC	Liver cancer tissues
HER2	human epidermal growth factor receptor 2
HGSC	high-grade serous carcinoma
ICG	indocyanine green
IDC	invasive ductal cell carcinoma
IDH	isocitrate dehydrogenase
iMRI	intraoperative magnetic resonance imaging
LAMs	lipid-associated macrophages
LC-MS	Liquid chromatography MS
LCFs	long-chain fatty acids
LDA	linear discriminant analysis
LMJ-SSP-MS	liquid microjunction surface sampling probe MS
LPC	lysophosphatidylcholine
LPE	lysophosphatidylethanolamine
MALDI-MS	Matrix-Assisted Laser Desorption/Ionization
MS	mass spectrometry
MUFA	monounsaturated FA
NAA	N-acetylaspartate
NSCLC	non-small-cell lung cancer
OC	ovarian cancer
OCCC	ovarian clear cell carcinoma
OSCC	oral squamous cell carcinoma
PAs	phosphatidic acids
PCA	Principal component analysis
PCs	phosphatidylcholines
PDAC	pancreatic ductal adenocarcinoma
PEs	phosphatidylethanolamines
PESI-MS	Probe electrospray ionization mass spectrometry
PI	phosphatidylinositol
PIRL-MS	Picosecond InfraRed Laser mass spectrometry
PLK1	polo-like kinase 1
PS	phosphatidylserine
PTC	papillary thyroid carcinoma
PTEN	phosphatase and TENsin homolog
PUFA	polyunsaturated FA
p53	tumor protein p53
REIMS	Rapid Evaporative Ionization Mass Spectrometry
SCC	squamous cell carcinoma SCC
SFA	saturated FA
SSI	Sonic Spray Ionization
SVM	Support Vector Machine
TCP	percentage of tumor cells
TSR	tumor stroma ratio
UHPLC	Ultra-High-Performance Liquid Chromatography
VLCF	Very-long-chain fatty acids
WALDI	water-assisted laser desorption/ionization
