# Supplementary material for: Intraoperative Mass Spectrometry in Oncology: Technologies, Clinical Applications, and Challenges
Source: Molecules. 2026 Apr 15;31(8):1287. doi: 10.3390/molecules31081287 (PMC13118378; doi:10.3390/molecules31081287)
Supplement: Supplementary file 1 [file molecules-31-01287-s001.zip › molecules-4199259-supplementary.pdf]

Supplimentary files

Table S1. Clinical implementation of intraoperative MS techniques based on reported studies.

Method	Cancer type	Implementation context	Sample size	Key performance	Clinical applicability	Ref.
**DESI MS**	Gliomas	Ex vivo (fresh-frozen human glioma biopsies, analyzed as smears)	64 biopsies (38 core + 26 margins)	-Sensitivity 88–94%; -Specificity 100%; -Accuracy 95–97%; -Concordance 94–96% (vs intraoperative assessment)	-Intraoperative detection of IDH mutation -Assessment of tumor infiltration at margins (via 2HG levels) -Support for surgical decision-making -Guidance of extent of tumor resection	[114]
**DESI MS**	Gliomas	Ex vivo analysis of intraoperative human tissue smears	-Patients: 10 (surgical cases) -Biopsies / smears: 73 biopsies (tissue smears) -44 margin samples (from total)	-detection of glioma: 93% sensitivity and 83% specificity - no numerical concordance (%) reported	-Intraoperative assessment of tumor margins -Detection of glioma infiltration -Estimation of tumor cell percentage (TCP) -Identification of IDH mutation (via 2HG detection) -Guidance of surgical resection	[76]
**DESI MS**	Gliomas	Ex vivo (intraoperative human tissue biopsies)	49 patients; 203 biopsies; 272 smears (from those biopsies)	Sensitivity/specificity/accuracy for: -IDH mutation status: 89% / 100% / 94%; -TCP (NAA method): 91% / 76% / 83%; -TCP (lipid deconvolution): 76% / 85% / 81%; -Disease status (PCA-LDA: 63% / 83% / 74%; -no overall concordance % reported	-Intraoperative glioma diagnosis -IDH mutation status determination -Estimation of tumor cell infiltration (TCP) -Assessment of surgical margins (residual tumor) -Support for surgical decisions (extent of resection)	[77]

**DESI MS**	Gliomas	Ex vivo (human brain tissue samples and smears analyzed outside the body)<br><br>Rapid (5 mins) biomarker identification in OR	32 human brain tissue samples (extracts) ; 29 samples used as smears for DESI-MS analysis	Sensitivity / Specificity/Accuracy, reported for DESI-MS ion abundance ratios:<br>- Positive ion mode ratios: >90%/>80%/>85%<br>-Negative ion mode (hexol ratio): 92% /100%/ 97%<br><br>Concordance not reported	-Rapid intraoperative tissue diagnosis of glioma<br>-Differentiation between normal brain parenchyma and glioma<br>-Improvement of tumor margin identification<br>-Support for maximizing tumor resection and personalized therapy	[115]
**DESI MS**	Oligodendroglioma, astrocytoma, and oligoastrocytoma	Ex vivo (frozen human glioma tissue sections obtained from samples collected duting surgery)	36 total samples	-No sensitivity/specificity reported;<br>- Overall recognition capability (≈ 99%) and cross-validation (≈ 97 – 99%) of machine learning model (SVM) for the classification performance of glioma subtype, grade, and tumor cell concentration<br>-Concordance with histopathology diagnosis ~81%	-Rapid classification of gliomas based on lipid profiles<br>-Near real-time intraoperative tissue characterization<br>-Differentiation of tumor vs normal/infiltrated brain tissue<br>-Assessment of tumor heterogeneity and margins<br>-Support for surgical decisions	[116]
**DESI MS**	Normal and pathological (glioma) brain tissue samples	Ex vivo tissue sections analyzed after surgical collection	39 patients; hundreds of regions of interest (ROIs)(e.g., 223 gray matter, 98 white matter, 185 glioma)	sensitivity ~93–95%, specificity ~96–98% (depending on ion mode)	Diagnostic tool for brain tumor classification and intraoperative guidance, including margin assessment and support for histopathology	[118]
**DESI MS**	Normal and pathological (glioma) brain tissue samples	Ex vivo (resected neurological tissue smears)	29 patients: 29 samples (tissue smear/ patients)	-No sensitivity/specificity reported;<br>-correspondence between TCP estimated by DESI-MS and pathology is 1:1	-Intraoperative diagnosis,<br>-tumor classification, -TCP estimation, -complement to histopathology,<br>-faster than frozen sections	[90]
**DESI MS**	Gliomas	Ex vivo (resected glioma tissue sections and intraoperative biopsies)	35 glioma specimens; + 2 intraoperative cases	-Sensitivity: 100% (all mutant detected); -	-Intraoperative detection of 2-HG and IDH	[119]

				Specificity: 100% (no false positives, no signal in wild-type cases); -Concordance: not quantified	mutation status identification, (DESI-MS (profiling), -tumor margin assessment (DESI-MS imaging (2D), -surgical guidance	
**DESI MS**	Gliomas and meningiomas	Ex vivo analysis of intraoperatively obtained human tissue	-banked samples: 55 in total (training: 21 gliomas ,11 meningiomas ; validation : 15 gliomas. 8 meningiomas) -32 surgical specimens (5 patients)	-concordance with histopathology (100% in validation set); cross-validation accuracy 99.7%; -no sensitivity/ specificity reported	-intraoperative tumor diagnosis, -classification, -grading, -tumor margin assessment	[39]
**DESI MS**	Gliomas, meningiomas, and pituitary tumors	Ex vivo (cryosectioned human brain tissues and tissue smears)	58 patients; multiple regions/ROIs analyzed (e.g., 223 gray matter, 66 white matter, 158 glioma, 111 meningiomas, 154 pituitary tumors)	-Differentiation: brain parenchyma vs glioma : Sensitivity: 97.4%/specificity 98.5% -Differentiation: tumor types (glioma, meningioma, pituitary) : Sensitivity: up to 99.4% / specificity 99.7%	-Tumor classification, -differentiation from normal tissue, -infiltration detection, -intraoperative guidance	[121]
**DESI MS**	Glioblastoma	Ex vivo (tissue sections from human U87 glioblastoma xenograft grown in mice)	15 tissue sections (number of tumors/animals not clearly specified)	Sensitivity. Specificity, accuracy and concordance – not reported	-3D mapping of tumor heterogeneity, -correlation with hypoxia and lipid metabolism (research / proof-of-principle)	[122]
**iKnife**	Different glioma subtypes	Ex vivo analysis of human surgical tissue	36 specimens (36 patients) (with 124 REIMS-sampling points: 31 normal, 93 tumor)	-Overall classification (multi-class glioma types): overall accuracy: 87.90% (cross-validation) -High-grade glioma (GBM percentage model) Accuracy: 97.04% Sensitivity: 99.3% Specificity: 100%	-Real-time intraoperative tissue characterization -Differentiation of glioma types and grades -Tumor margin detection and quantification of tumor cell percentage -Support for surgical decisions	[59]

				-Low-grade glioma (separate model)<br>Accuracy: 96.49%<br>False negative rate: 6%<br>False positive rate: 0%<br>-Astrocytoma grade II percentage model<br>Accuracy: 75%<br>Sensitivity: 97.5%<br>Specificity: 50%		
**iKnife**	Brain, liver, lung, breast, gastric and colorectal tumors	-healthy tissue - in vivo<br>-cancerous tissue - both in vivo and ex vivo (for validation)	large, multicenter study: 393 cases (divided in subgroups)	- Overall accuracy ≈ 96% diagnostic accuracy (cross-validation)<br>-For tumor vs healthy differentiation: sensitivity ≈ 97% specificity ≈ 96%	-intraoperative real-time tissue identification<br>-differentiation: healthy vs tumor tissue<br>-surgical resection guidance<br>-tumor margin delineation<br>-reducing reliance on extemporaneous examination	[38]
**PIRL MS**	Medulloblastoma	Ex vivo (murine xenograft tumors from human medulloblastoma cell lines)	19 tumors (6 different human medulloblastoma cell lines); 194 spectra	-Accuracy in subgroup determination: 98%<br>-Cross validation: 94%<br>-No sensitivity / specificity/concordance values reported	-rapid subgroup classification of medulloblastoma subgroups (SHH vs Group 3);<br>-potential intraoperative application-proof-of-principle (5–10 seconds analysis)	[124]
**PIRL MS**	Medulloblastoma	Ex vivo human tumor specimens (banked, frozen tissue)	113 tumors (72 training + 41 validation); 124 sampling events **(spectra)** in validation	-accuracy (classification rate): up to 98.9%<br>-no sensitivity/specificity<br>-concordance: implied vs molecular gold standard, not expressed numerically	-Rapid intraoperative molecular classification of medulloblastoma subgroups<br>-Real-time decision support during surgery (~10 seconds)<br>-Potential personalization of surgical strategy	[78]
**PIRL MS (primary) DESI MS**	Mouse brain tissue	Ex vivo (mouse brain tissue, xenograft tumors, tissue sections)	4 orthotopic xenograft tumours (Med8A and DAOY); additional 69 ROI spectra	-No sensitivity / specificity values reported	-Cancer margin assessment,<br>-tumor vs	[125]

**(comparative)**				- concordance: reported qualitatively (with pathology), no %	healthy classification, -molecular imaging of tissue heterogeneity; -building cancer m/z signature libraries; -potential intraoperative application (ex vivo)	
**PIRL MS**	Brain pediatric tumors: Medulloblastoma pilocytic astrocytoma ependymoma	Ex vivo analysis of human tumor tissue (banked, frozen specimens) - retrospective patient tissue study	156 banked specimens	Sensitivity and specificity for tumor-type classification (multi-class): 96.41% and 99.54% - based on molecular profiling data as the reference standard (94.39% and 98.78% using 18 biomarkers panel).	-rapid (10 s) intraoperative molecular classification of tumors - differentiation of pediatric brain tumor types and subtypes -support for surgical decision-making	[51]
**PIRL MS**	Metastatic carcinomas, meningioma, schwannoma, myxopapillary ependymoma, neurofibroma, paraganglioma, hemangiopericytoma	Ex vivo human tissue samples (biobanked, frozen)	319 total samples (metastatic carcinoma: 62 ,meningioma: 97, schwannoma: 106, others (ependymoma, neurofibroma, etc.): remaining samples); 182 for evaluation; 60 blind validation	-Sensitivity: ~92–93% -Specificity: ~96–97% - Concordance: ~92.34% (spatial concordance across sampling points)	-Rapid (~10 s) intraoperative diagnosis of spinal tumor types - Non-subjective alternative to neuropathology consultation -Decision support for surgical strategy	[80]
**iKnife**	Breast cancer	Ex vivo human tissue samples (burns from excised specimens)	320 skin burns from 51 patients (129 tumoral and 191 non-tumoral); 144 breast tissue burns from 11 patients (41 tumoral and 103 non-tumoral) Additional prospective test set: 44 burns (8 tumor, 36 normal) from 3 patients	Accuracy – 92%, sensitivity – 88%, specificity – 92-96%	Real-time breast cancer margin detection based on metabolomic signature	[127]
**PIRL MS**	Breast cancer	Ex vivo (resected human breast specimens)	6 human breast samples (4 infiltrating ductal carcinoma and 2 non-tumoral)	Short MS analysis; automated real-time image; avoid contamination for MS characterization	-Mapping of adipose/non-adipose tissue belongs to healthy and cancerous breast tissue -Guides MS sampling,	[128]

					-avoids fat contamination, -improves diagnostic efficiency and signal quality	
**iKnife**	Brest cancer	Ex vivo cancerous tissue analysis and peri-tumor stromal tissue analysis, future in vivo implementation	Sample tissues from 72 patients	Tissue type classification accuracy – 92.3%; tumor border stroma and remote border stroma accuracy 86%-87%	Precise surgical guidance for tissue surrounding malignant tumors	[129]
**iKnife**	Breast cancer	Ex vivo and In vivo implementation	17 cancer samples and 17 normal breast tissue	Sensitivity – 90.9%, specificity – 98.8%, accuracy – 95.8%, data acquisition and analysis in 1.8 seconds	Separation of breast tissue types, identification of resection margins	[56]
**DESI MS**	Breast cancer	Ex vivo (mouse xenograft tumors from human breast cancer cells)	At least 2 mice (exact number not clearly specified)	-concordance qualitative (agreement with MRI, histopathology)<br><br>-no sensitivity / specificity values reported	-Tumor margin identification -Mapping intratumoral heterogeneity (vasculature, necrosis) -Visualization of contrast agent distribution -Complement to MRI imaging -Potential intraoperative tumor mapping without prior MS profiles	[130]
**DESI MS**	Breast cancer	Ex vivo (xenograft tumor tissues from mice; slices and smears)<br>In vivo MR imaging	4 female mice inoculated with triple negative cancer cells	-no sensitivity / specificity values reported -concordance: qualitative (with pathology), no numerical values	-Necrotic breast cancer profile -Rapid intraoperative detection of necrosis, -improved diagnosis speed, potential surgical guidance	[131]
**DESI MS**	Breast Cancer	Ex vivo (animal-derived tumor tissues, xenograft model)	Triple negative breast cancer cells inoculated in female mice: 12 tumor slices, 12 smear preparations; 24–26 ROIs	-No sensitivity values (numerical) reported - No specificity reported -Concordance is qualitative/statistical (PCA), not numerical	Rapid cancer typing (intraoperative use, <1 min analysis, faster than histology)	[132]

				between DESI-MS profiles of tissue sections and tissue smears		
**MasSpec Pen**	Breast, lung, thyroid, and ovary cancers	Ex vivo analysis, future in vivo application	253 human tissue samples: 95 lung, 57 ovary, 56 thyroid, 57 ovary and 45 breast samples	Cancer prediction sensitivity – 96.4%, specificity – 96.2%, accuracy – 96.3%	Identification of tumor extension for accurate negative margin excision	[50]
**DESI MS**	Breast cancer	Ex vivo (mouse xenograft tumor sections from human breast cancer)	2 mice (2 tumors); multiple sections (not specified)	-no sensitivity / specificity values reported -concordance: quantitative correlation with tissue density ($R^2 \approx 0.36–0.82$)	-Methodological: improves interpretation of DESI-MS data and biomarker reliability; -preclinical	[133]
**DESI MS**	Breast cancer	In vitro (2D cell cultures + 3D spheroids) - for biomarker detection	Breast cancer and non-cancer cell lines - multiple cell lines (~9);5 sample spots per cell line; >200 lipids per cell line	Detection of lymph node metastasis; cancer staging; discrimination between tumor and normal tissue (potential clinical tool, requires validation) qualitative (PCA-based classification of cell lines)	Identification of lipid biomarkers (over 200 lipid species have been identified/cell lines; -classification by metastatic potential, disease state, HER2/p53; -research/ potential clinical relevance	[134]
**DESI MS**	Breast and thyroid cancer	Ex vivo analysis of samples (banked frozen human lymph node tissues), need for statistical analysis to confirm DESI MS findings, and to further implement it clinically	42 tissue samples: Lymph node metastatic tumors (16 breast cancer, 8 thyroid cancer) and 18 normal lymph nodes	-no sensitivity / specificity values reported -concordance: qualitative (correlation with histopathology)	-rapid detection of lymph node metastases of breast and thyroid cancer based on lipid and metabolite profiles<br>-cancer staging; -discrimination between tumor and normal tissue (potential clinical tool, requires validation)	[135]
**DESI MS**	Retinoblastoma cell cultures	In vitro (cancer and normal cell lines: MCF-7 (breast cancer) and WERI-RB1 (retinoblastoma), MIO-M1 (normal)	No clear total number of analyzed samples/specimens is provided	-no sensitivity / specificity/concordance values reported	-Monitoring therapy response; -drug screening; -lipid biomarker discovery (preclinical research tool)	[136]

**DESI MS**	Colorectal cancer	Ex vivo analysis of a specimen, PCA is unsuitable for the identification of clusters of cancerous tissue, and needs a neural-network-based technique	10 samples, 52 frozen tissue sections	-no sensitivity / specificity values reported<br>-concordance between DESI-MS and UPLC-ESI-MS lipid profiles: correlation coefficient ~0.7	-identification of tumor-specific clusters for different tissue regions<br>-lipid profiling, tissue characterization,<br>-potential histological classification and future clinical applications	[137]
**DESI MS**	Colorectal cancer	Ex vivo sample analysis, data analysis with PCA and LDA	31 primary colorectal adenocarcinoma and 40 liver metastases of colorectal carcinoma samples	95% diagnostic agreement with histology and 90% with molecular biology	Diagnostic lipid constituents of tissue-specific species	[138]
**iKnife**	Colorectal cancer	Ex vivo analysis of specimens, LDA and PCA statistical analysis, and future in vivo applications	28 patients undergoing surgery for colorectal cancer, 2 of which had adenoma	94.4% overall accuracy for detection cancer vs adenoma with 78.6% sensitivity and 97.3% specificity	High precision of histological features in poor prognosis colorectal cancer patients	[58]
**DESI MS**	Esophageal cancer	Ex vivo specimen analysis, statistical PCA analysis	Samples of 10 patients with esophageal adenocarcinoma	-concordance between DESI-MS and UPLC-ESI-MS lipid profiles: correlation coefficient ~0.7<br>-no sensitivity / specificity values reported	-Lipidomic profiling,<br>- tissue characterization,<br>-potential for histological classification and future clinical applications	[139]
**MasSpec Pen**	Pancreatic cancer	Ex vivo sample analysis and in vivo application. Comparison between results	157 banked human tissues (pancreatic ductal adenocarcinoma, pancreatic and bile tissues)	91.5% accuracy with histology, 95.5% sensitivity, 89.7% specificity	High performance in intraoperative use to guide surgical decision	[65]
**DESI MS**	Renal cancer	Ex vivo (human tumor and adjacent normal tissue sections)	48 pairs (tumor + adjacent normal tissue) ; 96 tissue samples (sections), paired	-concordance: qualitative agreement with histopathology (no quantitative metrics)<br>-no sensitivity / specificity values reported	-Complementary diagnostic tool;<br>-improves tumor vs normal discrimination; - potential to enhance histopathology	[140]
**DESI MS**	Bladder cancer	Ex vivo sample analysis, orthogonal projection to latent structures (O-PLS), treated partial least-square discriminate analysis (PLS-DA) statistical analysis.	20 pairs of human bladder cancer and adjacent tissue	5% error classification rate and 12% misclassification rate	Surgical identification of tumor margins	[141]

**DESI MS**	Canine bladder cancer	Ex vivo (canine bladder tissue sections) sample analysis, PCA statistical analysis	Transitional cell carcinoma tissue samples from 4 dogs (pairs of cancerous and normal tissue)	--No sensitivity/specificity/ reported -concordance: Not reported (qualitative agreement with H&E only)	-potential for use in a diagnostic capability (tumor vs normal discrimination) using lipid profiles; -proof-of-concept diagnostic tool	[142]
**DESI MS**	Renal cancer	Ex vivo specimen analysis, differentiation between renal cancer cell types	81 banked frozen human tissue samples (uninvolved 20 kidney with cortex and/or medullar, 15 renal oncocytoma and 46 renal cell carcinoma samples) - 71 samples analyzed (after quality control)	-No sensitivity/specificity/concordance reported -Accuracy (87–100%), recall (~99.6%), ROC AUC = 1	-Tumor vs normal discrimination; -benign vs malignant differentiation; -RCC subtype classification; -potential clinical diagnostic tool	[143]
**DESI MS**	Renal cancer (papillary and clear renal cell carcinoma -RCC)	Ex vivo specimens (human kidney tissue sections) analysis, partial least square discriminate analysis (PLS-DA) statistical analysis	11 matched pairs of papillary RCC and adjacent normal tissue and 9 matched pairs of ccRCC and adjacent normal tissue	--No sensitivity/specificity/concordance reported<br><br>-Misclassification rates (7.8–23.7%)	-Tumor vs normal discrimination; -subtype classification (papillary vs clear cell RCC); -potential diagnostic tool -the use of multivariate statistical methods increases the confidence of this diagnosis	[144]
**DESI MS**	Prostate cancer	Ex vivo sample (human prostate tissue sections) analysis, PCA statistical analysis	68 samples of human prostate cancer and normal tissue ((34 patients)	-No sensitivity/specificity reported<br><br>-concordance: ~94% (64/68 samples)	-Cancer vs normal differentiation; -detection of PIN; (precancerous lesions) -biomarker discovery (cholesterol sulfate); -potential diagnostic tool	[145]
**DESI MS**	Prostate cancer	Ex vivo (human prostate tissue, frozen sections) sample analysis, possible future in vivo application	54 normal and malignant prostate tissue specimens (36 training + 18 validation) + 10 mixed	-No sensitivity/specificity reported<br><br>-concordance: ~89–94% agreement with pathology	-large heterogeneity in lipidomic profiles of prostate cancer patients -Rapid cancer diagnosis (~1 min vs ≥20 min	[146]

					histology); -intraoperative margin assessment; -surgical guidance; -potential biopsy use	
**iKnife**	Skin cancer (basal cell carcinoma)	Ex vivo sample analysis, Bayesian neural network, and two baseline models are trained on these data to perform classification as well as uncertainty estimation	693 spectra of cancer and healthy samples from 91 patients - basal cell carcinoma resection	-accuracy (75.2%), -sensitivity (74.1%) -reduced specificity compared to filtering, -data-centric methods effective in yielding high-quality datasets to enhance classification performance of models	-first study that applies uncertainty estimation to inform model training and deployment for tissue recognition in cancer surgery	[61]
**DESI MS**	Oral tongue squamous cell carcinoma	Ex vivo sample analysis, PCA-LDA statistical analysis, and accurate differentiation of normal to cancerous tissue	46 SCC and normal tissue samples	PCA-LDA accuracy rates of 95% for SCC versus normal and 93% for SCC, adjacent normal and normal.	-may be useful in the diagnosis of oral tongue squamous cell carcinoma; -approx. 20 min. per sample not compatible with point-of-care use	[147]
**SpiderMass**	Oral tongue squamous cell carcinoma	In vivo real-time analysis of different areas: tumor, nontumor, dysplasia, peritumoral, the use of PCA-LDA for tumor classification, complementary MALDI MSI was used for ex vivo validation on histologically annotated tissue sections.	14 SCC samples	sensitive and specific enough to detect subtle changes within the tumor and nontumor tissues as well; accuracies ranging from 71.7% to 83.2% depending on the type of method used	-constructing the SpiderMass databank, -prospective, noninterventional pilot study	[84]
**DESI MS**	Mouse xenograft tumors from the human FaDu cell line	In vivo (mouse), ex vivo (tumor tissue), in vitro (cell cultures)	3 independent mouse xenograft tumours from the human FaDu cell line - human pharynx squamous cell carcinoma line FaDu and human primary head and neck carcinoma-	-No sensitivity/specificity reported<br><br>-concordance: ~5% agreement with pathology	-determination of DESI-MS profile of human pharyngeal squamous cell carcinoma using mouse xenograft models from the established FaDu cell line -Rapid tumor stroma ratio	[148]

					(TSR) determination; -prognostic value; -intraoperative decision support	
**MasSpec Pen**	Ovarian cancer	Ex vivo sample analysis, future in vivo applications	160 ovarian frozen tissue samples	High performance for high-grade serous carcinoma - sensitivity, 96.7%; specificity, 95.7%; Variations in the mass spectra from normal tissue, low-grade, and high-grade serous OC;	MasSpec Pen, together with machine learning, provides robust molecular models for serous OC prediction - rapid and accurate OC diagnosis	[63]
**DESI MS**	Ovarian cancer	Ex vivo sample analysis, rapid diagnosis, future in vivo application	78 total samples - 15 normal ovarian tissues, 15 borderline ovarian tumors (BOT), and 48 high-grade serous carcinoma (HGSC) samples	-No sensitivity/specificity reported -Performance expressed as agreement (%: 96.4% overall agreement (HGSC vs normal) and 96.2% overall agreement (tumor vs normal) and accuracy (93%: (HGSC vs BOT discrimination) -Predictive markers of cancer aggressiveness were identified	Markers identified-FFAs, metabolites, ceramides, glycerophosphoglycerols, cardiolipins, and GPCs; -Diagnosis of ovarian cancer; -discrimination between tumor types; -prediction of aggressiveness; -biomarker discovery; -improved clinical management	[149]
**iKnife**	Ovarian cancer	In vivo and ex vivo use, PCA and LDA statistical analysis	198 patients - 335 tissue samples	OC classification vs separate normal tissues (97·4% sensitivity, 100% specificity); Borderline tumors readily distinguishable from OC (sensitivity 90.5%, specificity 89.7%) excellent OC detection (100% accuracy) Histological agreement	The REIMS iKnife distinguishes gynaecological tissues by analysing mass-spectrometry-derived lipidomes from tissue diathermy aerosols; may improve surgical care when histology is unknown, leading to personalised operations	[57]

				between iKnife and histopathologist was very good	tailored to the individual	
**DESI MS**	Thyroid cancer: PTC (papillary thyroid carcinoma) and FTC (follicular thyroid carcinoma)	In vivo (preoperative fine-needle aspiration- FNA-samples) and ex vivo (tissue sections + some FNA samples) applications, high sensitivity and specificity with high accuracy as well	206 frozen human thyroid tissue samples + 69 prospectively collected clinical fine-needle aspiration (FNA) smears	-PTC: sensitivity of 92,-96% and specificity of 91-94% -FTC: sensitivity of 65.2-100%, specificity of 80-91%, (tissue vs FNA dataset) -Accuracy (per-sample agreement with pathology): 93% -for benign vs. PTC model – both tissue and FNA datasets -Accuracy (per-sample agreement with pathology): 80-89% -for benign vs. FTC model – depending on the dataset	-diagnose thyroid lesions from FNA samples using metabolic data collected via DESI-MS imaging; -direct analysis of fine needle aspiration smears without any sample modification -preoperative diagnosis of thyroid nodules, especially indeterminate FNAs; -improves clinical management	[150]
**DESI MS**	Thyroid cancer	Ex vivo sample analysis, further studies needed before in vivo applications	45 frozen human thyroid tissue specimens -	-no sensitivity / specificity/concordance values reported	-first study to report abnormal expression and composition of lipids such as cardiolipins in human thyroid tumor tissues; -diagnosis of thyroid tumors; -potential use in FNAB; -biomarker identification; -therapeutic targets	[151]
**CUSA MS**	Brain cancer - astrocytomas, meningeomas, metastatic brain tumors	Ex vivo + post-mortem human samples; animal tissues (porcine); not in vivo PCA LDA statistical analysis, future in vivo applications	284 spectra (number of patients/samples not specified) Unclear number of human brain cancer samples	-no sensitivity / specificity/concordance values reported -faster tissue identification than standard methods (23s vs 30-40 min)	Possibility of coupling ultrasonic surgical techniques with online MS analysis; -not yet been tested for in vivo tissue identification	[41]

					-tumor vs healthy differentiation; -surgical guidance	
**SpiderMass**	Skin cancer	Ex vivo / in vivo/ **in vitro (method-focused study)**	Unclear number of skin tissue samples	-no sensitivity / specificity/concordance values reported	-technology platform for in vivo, real-time MS-guided diagnosis/surgery (WALDI ionization) -LDA + PCA - data separation and building of classification models -Not yet clinically validated (protocol-level work)	[67]
**SpiderMass**	Frozen rat brain tissue	Primarily in vivo (+ ex vivo validation) Design implementation of the technique combined with a robotic arm for high accuracy and for 3d topographical imaging, PCA-LDA statistical analysis	Unclear number of fresh frozen rat brain samples	-no sensitivity / specificity/concordance values reported	-Robot-assisted in vivo imaging; -surgical navigation (proof-of-concept) --future clinical use in oncology surgery as an autonomous device, in contrast to coupling the probe to robot-assisted surgery devices	[68]
**SpiderMass**	Human skin	In vivo (human skin) + Ex vivo (biopsies, tissues)	10 subjects (in vivo) 2 biopsy samples (ex vivo, same patient)	-no sensitivity / specificity/concordance values reported	-First time application (clinical) of SpiderMass – describing the instrument advantages and disadvantages -Real-time molecular profiling, -tumor differentiation, -potential guided surgery and diagnostic applications	[40]
